# Damages at the nanoscale on red blood cells promoted by fire corals

**DOI:** 10.1038/s41598-019-50744-6

**Published:** 2019-10-04

**Authors:** Ana R. Díaz-Marrero, Miriam C. Rodríguez González, Alberto Hernández Creus, Adriana Rodríguez Hernández, José J. Fernández

**Affiliations:** 1 Instituto Universitario de Bio-Orgánica Antonio González (IUBO AG), Centro de Investigaciones Biomédicas de Canarias (CIBICAN), Universidad de La Laguna (ULL), Avda, Astrofísico Francisco Sánchez 2, 38206 La Laguna, Tenerife Spain; 2 Departamento de Química, Área de Química Física, Instituto de Materiales y Nanotecnología (IMN), Universidad de La Laguna (ULL), Avda. Astrofísico Francisco Sánchez s.n., 38206 La Laguna, Tenerife Spain; 3 Departamento de Biología Animal, Edafología y Geología. UD Ciencias Marinas. Facultad de Ciencias (Sección Biología), Universidad de La Laguna (ULL), Avda. Astrofísico Francisco Sánchez s.n., 38206 La Laguna, Tenerife Spain; 4 Departamento de Química Orgánica, Universidad de La Laguna (ULL), Avda. Astrofísico Francisco Sánchez s.n., 38206 La Laguna, Tenerife Spain

**Keywords:** Imaging, Atomic force microscopy

## Abstract

The hydrocoral *Millepora alcicornis*, known as fire coral, biosynthesize protein toxins with phospholipase A2 (PLA2) activity as a main defense mechanism; proteins that rapidly catalyse the hydrolysis at the sn-2 position of phosphatidylcholine-type phospholipids of cellular membranes. This hydrolysis mechanism triggers a structural damage in the outer leaflet of the red blood cells (RBC) membrane, by generating pores in the lipid bilayer that leads to a depletion of the cellular content of the damaged cell. A secondary mechanism, tentatively caused by pore-forming proteins toxins (PFTs), has been observed. The use of atomic force microscopy (AFM) has allowed to visualize the evolution of damages produced on the surface of the cells at the nanoscale level along the time.

## Introduction

The hydrocoral *Millepora alcicornis* (Linnaeus, 1758) known as fire coral has an amphi-Atlantic distribution as evidenced by its presence in the Caribbean, Brazil (including Fernando de Noronha and Atol Das Rocas), Bermuda, Ascension Island^1^, Cape Verde Islands^2^, Canary Islands^3^ and Madeira Island^4^. In general, in tropical regions, *M*. *alcicornis* plays an essential role in the organization of coastal benthic communities^5^ and are well known for being a relevant reef builder as consequence of their large calcareous skeletons and for inflicting painful stings to humans^6^. The hydrocoral has high growth and rates of recruitment as well as high capacity to colonize natural and artificial substrates, such as rocks, dead calcifiers organisms and hull of ships^6–8^.

Temperature has been suggested to be one of the most important environmental factors controlling coral species distribution^9^, the arrival of coral species in new areas can have dramatical impacts on local ecological communities, which could lead to altered environmental conditions and novel interactions, competing with local endemic species such as *Oculina patagonia* in Mediterranean Sea^10^.

In Canary Islands *M*. *alcicornis* was registered for the first time in 2008, where 3 colonies were recorded in southeast of Tenerife Island, at 11°N of its previously known northernmost limit of distribution in Cape Verde Islands^3^. The arrival of *M*. *alcicornis* may be related with an extreme weather event registered in 2004 where 27 °C in surface temperature in Tenerife Island was recorded^3,11^. Genetic studies show than *M*. *alcicornis* arrived from Caribbean region and not from Cape Verde Islands^12,13^. From 2008 to the present a monitoring on this species has been done, observing an increase in the number of colonies and in its cover area^14^. Experimental studies on *M*. *alcicornis* from Tenerife Island shows its high tolerance to ocean acidification conditions and high increase in its cover are under rising temperature^15^. This suggests that under future climate change conditions *M*. *alcicornis* can become a winner species under climate changes scenarios.

**Figure 1:**
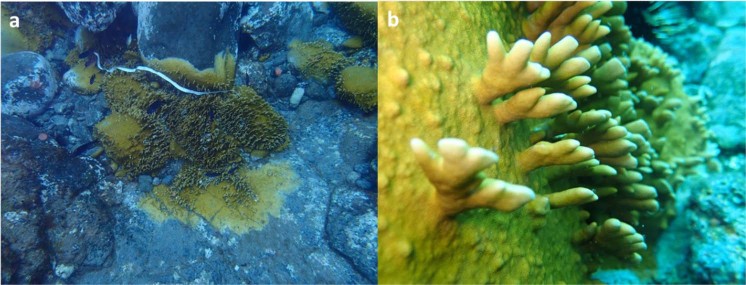
(**a**) Colonies and (**b**) detail of *Millepora alcicornis* in Tenerife Island.

Fire corals (hydrocoral, genus *Millepora*) (Fig. 1 and Supporting Information S1-S4) are known to cause painful stings, burning sensation and itching that last hours after contact and are a cause of discomfort for those who perform underwater, professional and recreational activities related to tourism. Several techniques have been used to isolate, purify and test the toxic contents of fire corals by demonstrating the existence of proteins, their hemolytic effects, and the presence of proteins of the phosholipase A2 (PLA2) type^16,17^. PLA2 is a known enzyme found in snakes and other venoms and body fluids^18^. In addition, PLA2s have been identified in other phyla including marine invertebrates, such as hard corals, fire coral, crown-of-thorns starfish, sea cucumber and marine sponges^16^. Thus, high PLA2 levels have been found in the hydrozoan fire coral *Millepora sp*. (735 U/g protein) and the stony coral *Pocillopora damicornis* (693 U/g) that it is associated with skin irritation upon contact. Chemically, PLA2 type enzymes cleaves glycerophospholipds at the sn-2 position and the reaction products are lysophospholipid and fatty acid^19–23^. Aside of PLA2 activity, pore-forming toxins (PFTs) have been described to target cell membranes (sphingomyelin, SM) leading to an osmotic imbalance and cell lysis by diffusion of cellular content through generation of pores^24–27^. Thus, this enzymatic activity could deconstruct cell membranes and especially those that come into direct or indirect contact with the coral proteins, with red blood cells being one of the possible targets involved.

Biological samples are complex systems that sometimes have to be monitored in their native state. Atomic Force Microscopy (AFM) is a suitable tool to image biological samples with molecular or submolecular resolution providing the corresponding topographical images. Nevertheless, the power of this technique is related with the possibility of measure these samples in air or in liquid environments which permits to obtain information of the samples in their native state. Thus, information at the nanoscale about topography, forces at molecular level or nanomechanical properties can be obtained for an almost unlimited number of proteins, cells or tissues^28,29^. In this manuscript a detailed description of the effects of the hemolytic-protein containing extract of fire corals in the RBC membrane has been done. Through a systematic study based on Atomic Force Microscopy, the degradation of the membrane has been visualized at the nanoscale level at different times, showing the appearance of damages and pores induced by the proteins present in the extract.

## Results and Discussion

**Figure 2:**
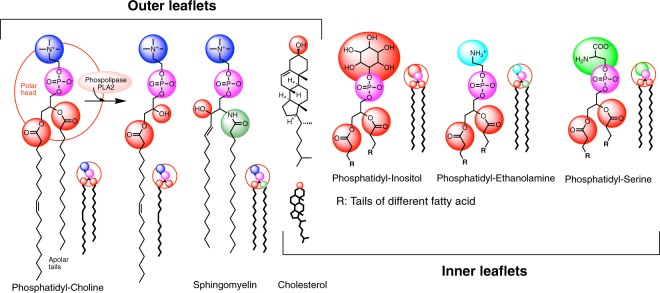
Major lipid constituents in red blood membrane structure. Outer leaflet: Phosphatidyl-Choline (PC), Sphingomyelin (SM, represented the C_16_ amide derivative) and cholesterol. Inner leaflet: Phosphatidyl-Ethanolamine (PE), Phosphatidyl-Serine (PS) and Phospho-Inositol (PI); R: aliphatic chain of different acyl fatty acid.

The red blood cell (RBC) membrane comprises a typical lipid bilayer. This lipid bilayer is composed of cholesterol and phospholipids in equal proportions by weight. Cholesterol is evenly distributed in the inner and outer leaflets, while five major phospholipids are asymmetrically disposed. Thus, in the outer leaflets phosphatidylcholine (PC) and sphingomyelin (SM) predominate; whereas phosphatidylethanolamine (PE), phosphatidylserine (PS) and phosphoinositol (PI) constitute the inner leaflets of the bilayer section (Fig. 2). This lipid composition determines the physical properties of the membrane, such as permeability and fluidity.

Inserted within the lipid bilayer, membrane proteins help to maintain the distribution of phospholipids^30^. The membrane proteins establish linkages with skeletal proteins and play an important role in regulating cohesion between the lipid bilayer and the membrane skeleton. As a result, the lipid bilayer, transmembrane proteins and a filamentous meshwork of proteins shape the membrane skeleton. Ankyrin proteins link the bilayer protein-complex membrane to the skeleton through the interaction of their cytoplasmic domains. The most abundant protein in this cytoplasmic system is spectrin, a cytoskeletal protein that lines the intracellular side of the plasma membrane.

**Figure 3:**
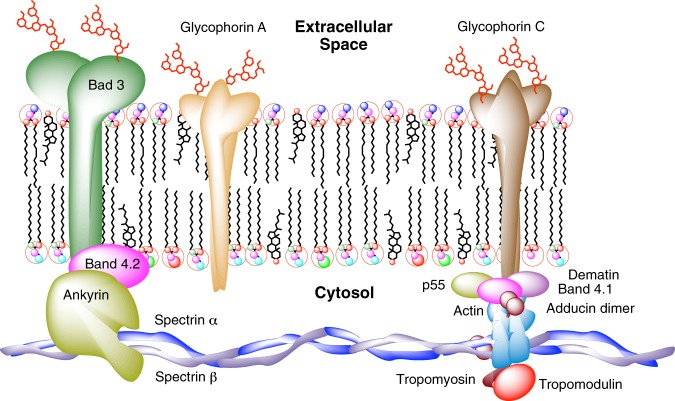
Schematic depiction of the principal components in the membrane structure and cytoskeleton of the red blood cells.

**Figure 4:**
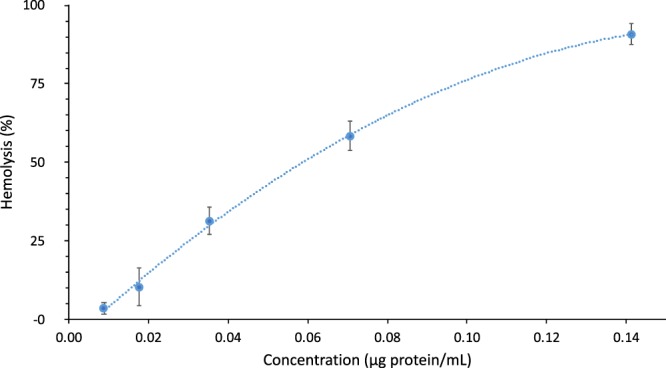
Concentration–response curve of the hemolytic activity induced by *Millepora alcicornis* aqueous extract on rat erythrocytes. Concentration represents protein content in the extract.

In RBCs, spectrin forms a hexagonal arrangement, forming a scaffold and playing an important role in the maintenance of plasma membrane integrity and cytoskeletal structure (Fig. 3). To study the effect of fire coral proteins, specimens of *Millepora alcicornis*, collected in the Canary Islands, were extracted in buffered aqueous solution at low temperature. The aqueous extracts were lyophilized and the protein concentration was quantified by the Bradford test, resulting in a content of 68.0 µg protein/mg lyophilizated extract. The hemolytic activity of the protein extract was evaluated against red blood cells obtained from the coccygeal artery of Sprange Dawley rats anesthetized with isoflurane, using EDTA (K3E) as anticoagulant. The obtained value of HU_50_ of 59 ng/mL showed a high hemolytic effect (Fig. 4). To unravel in detail how these hemolytic effects occur, an exhaustive and detailed study at the nanoscale using atomic force microscopy (AFM) techniques was undertaken.

**Figure 5:**
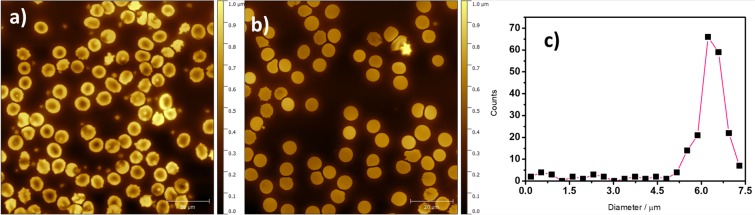
AFM images of 100 μm × 100 μm showing (**a**) discocytes and (**b**) planocytes; (**c**) diameter histogram for RBCs.

It is well known that RBCs can acquire different shapes such as discocytes, planocytes, stomatocytes, or echinocytes^31^. When control fresh rat blood samples were analysed, all these different morphologies were found (Fig. S5). Nevertheless, in the analysed samples, a vast majority of cells consisted of discocytes (Fig. 5a) and planocytes (Fig. 5b). In the large-scale images (100 μm × 100 μm) both structural shapes can be observed. Figure 5c shows the size histogram distribution obtained from different 100 μm × 100 μm images. The size of RBCs range between 5 and 7 μm, being the average diameter of most of the cells around 6 μm.

**Figure 6:**
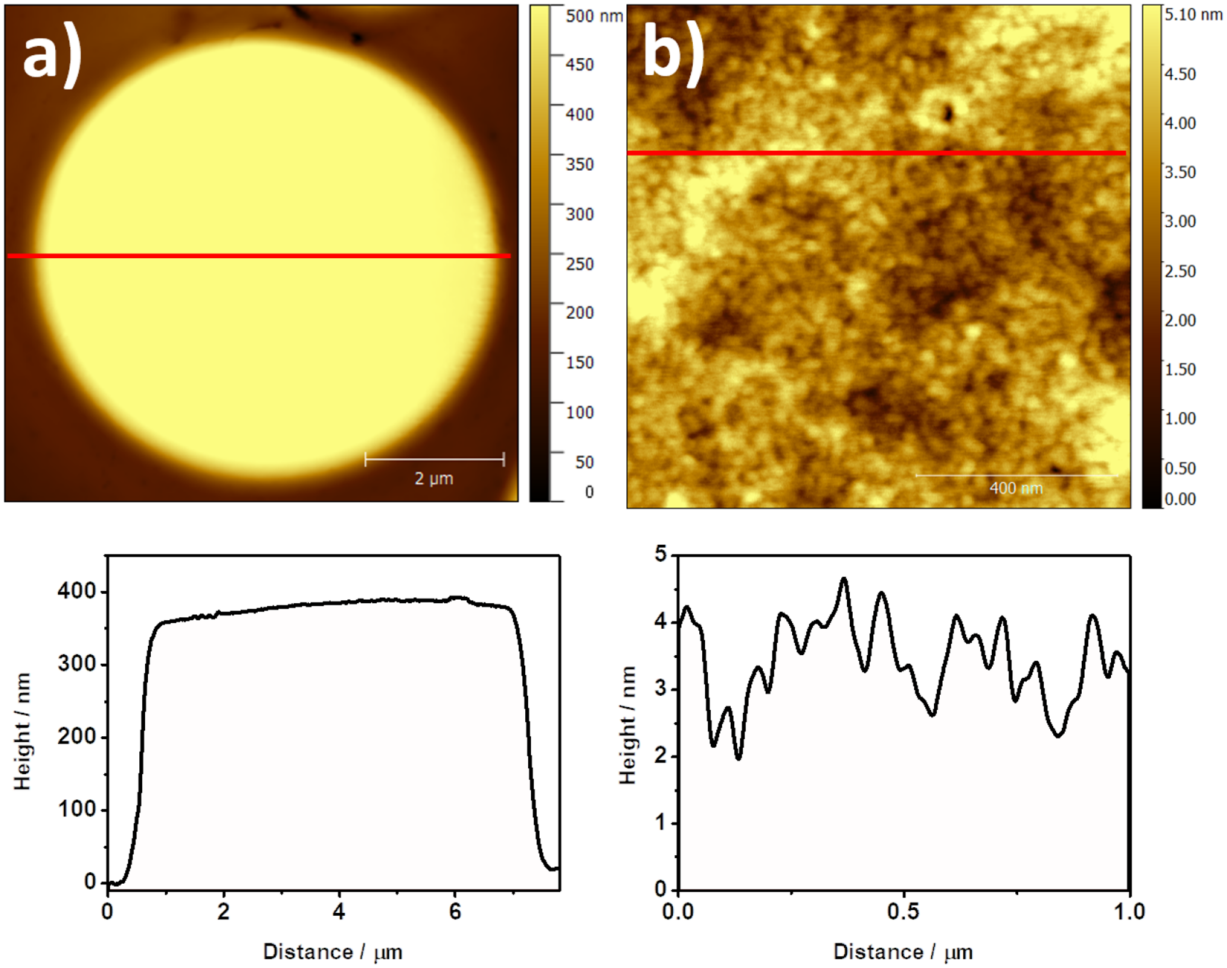
AFM images of (**a**) 8 μm × 8 μm, and (**b**) 1 μm × 1 μm of untreated RBCs.

A detail of the intact cells can be seen in Figs 6 and S6. These cells have a height around 300–400 nm as shown in the cross section. Moreover, when the structure is observed in a small scale (Fig. 6b) a very flat surface with a homogeneous distribution is found. The roughness of this surface is very low (≈ 0.83 nm) and it is related with a regular composition in the lipid bilayer that configures the outer part of the RBCs membrane^32,33^.

**Figure 7:**
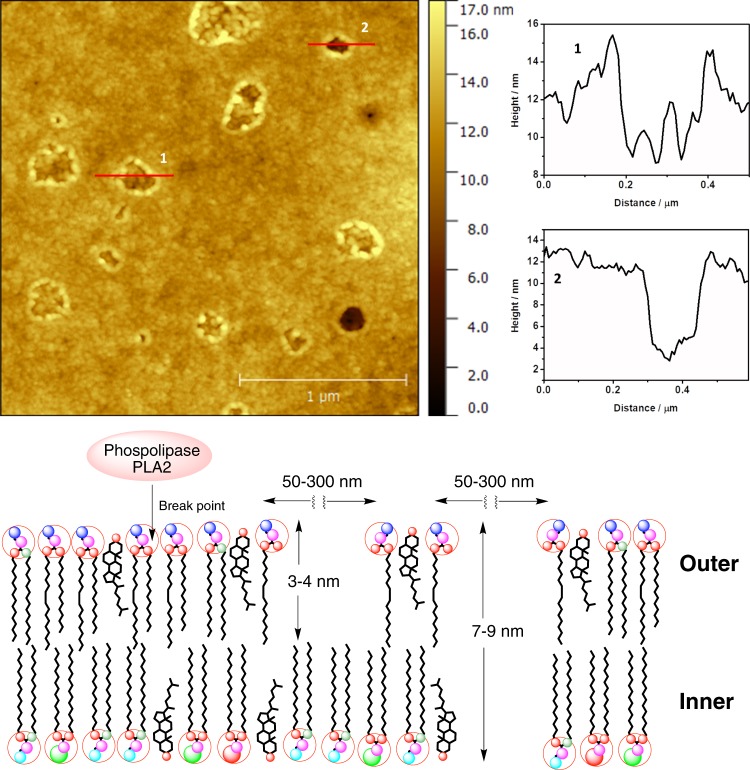
Top: AFM image of 3.5 μm × 3.5 μm of the surface of a RBC after 1 min in contact with the hemolytic protein extract from *M*. *alcicornis*, and cross sections **1** and **2**. Bottom: Schematic diagram of the initial membrane damage and formation of holes in the lipid bilayer in red blood cell.

To analyse the hemolytic effects of the protein extract of *M*. *alcicornis* on the surface of RBC membranes, rat erythrocytes were treated at different incubation times by adding an aliquote sample of the lyophilized extract regenerated in physiological saline solution. With the aim of producing an effective and fast hemolysis, a protein concentration of 100 μg/mL was employed. Figures 7 and S7–S9 show detailed AFM images of the response of rat blood cells after 1 min incubation. As images reveal, the surface of cells is highly covered by damages whose sizes range from 50 to 300 nm. At this initial stage, the maximum depth of the most representative defects is around 3 nm, whereas the least abundant damages, observed as dark spots, indicate a depth around 5–7 nm (Fig. 7). The presence of these holes can be rationalised as a consequence of the hydrolysis reaction between the membrane phospholipids catalysed by PLA2-type proteins from the coral extract^19,20^. PLA2-type proteins specifically trigger the rupture of the ester bond at the sn-2 position of the glycerol backbone of phosphatidylcholine residues in the outer leaflet of the cell membrane. The measured depths and the general aspect of these holes agree the expected distances for a degradation of the outer part of the bilayer, leaving exposed the inner part of this bilayer (Figs 7 and S7–S9).

**Figure 8:**
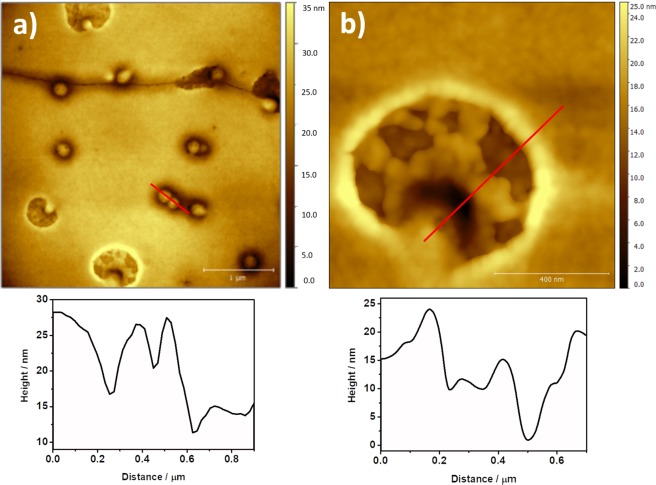
AFM images of (**a**) 4 μm × 4 μm and (**b**) 1 μm × 1 μm of the RBCs after 5 minutes in contact with the hemolytic protein extract, and cross sections.

When the incubation time in presence of the fire coral protein extract is increased up to 5 min, a different scenario is observed. In Fig. 8a vesicles homogenously distributed on the surface of the membrane can be found in addition to some bigger holes than those observed after 1 min of incubation. These vesicles have a size between 100 and 150 nm, and their presence has been reported as a consequence of the destabilization of the spectrin network found below the lipid bilayer^34^. In our case, this instability can be explained in terms of the lipid bilayer, as previously described^35^. In regard with the damages observed, Fig. 8 shows in detail that both the size and the height profile of the holes are different than those found in samples incubated for 1 min, and that a network corresponding to the spectrin protein complex can be seen (Figs 8B and S10–S12).

**Figure 9:**
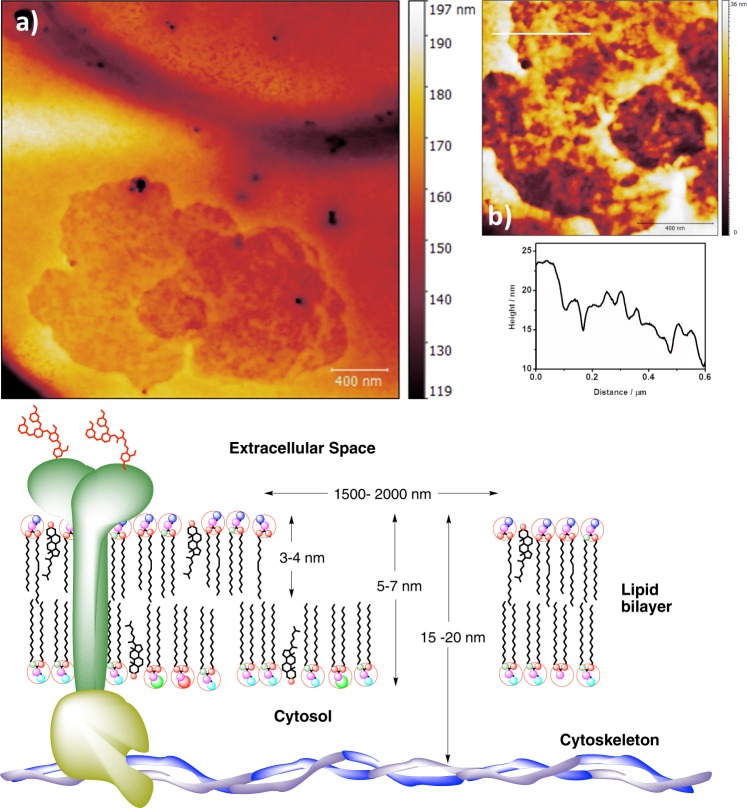
AFM images of (**a**) 2.5 μm × 2.5 μm and (**b**) 1 μm × 1 μm of the RBCs after 5 minutes in contact with the hemolytic protein, and cross section. Bottom: Schematic diagram of the total membrane damage and cytoskeleton in red blood cell.

In samples incubated for 5 and 10 min, the analysis of the surface of RBCs allowed to detect damages of higher dimensions that helped to explain the action mechanism of the hemolytic effect of fire coral proteins (5 min, Fig. 9; 10 min, Figs S13–S15). In this case, the network pattern observed in the hole seems to correspond with the underlying spectrin structure^30^. This fact can be better understood considering the cross section shown in Fig. 9b where two different levels can be distinguished. The first one (≈5 nm) can be associated to the average height of a lipid bilayer and the second one (≈15 nm), the deepest, corresponds to the spectrin network.

**Figure 10:**
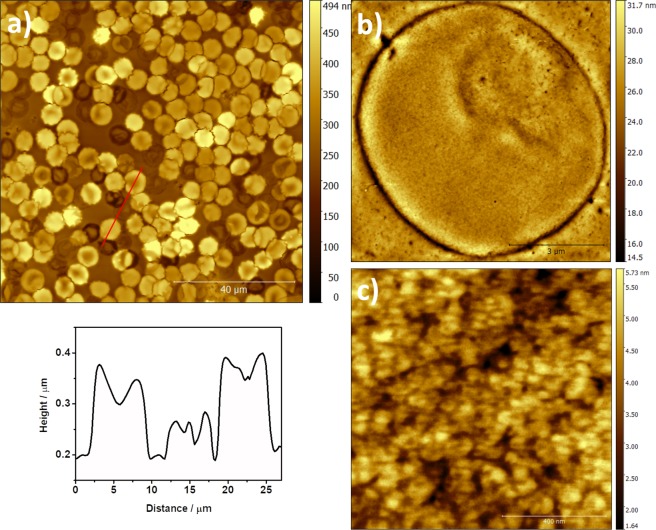
AFM images of (**a**) 100 μm × 100 μm; (**b**) 8 μm × 8 μm and (**c**) 1 μm × 1 μm detailed surface of the RBCs after 30 minutes in contact with the hemolytic protein.

Figures 10 and S16, S17 show the AFM images after 30 minutes of treatment. At this time, a large number of different states in the RBCs can be found, from a few of them almost intact to an increased population of completely deflated ones. The cross section shows the different heights for these diverse states. In the high-resolution image, a good illustration of the deflated RBCs can be observed with a less organized nanostructure when compared with the initial surface.

**Figure 11:**
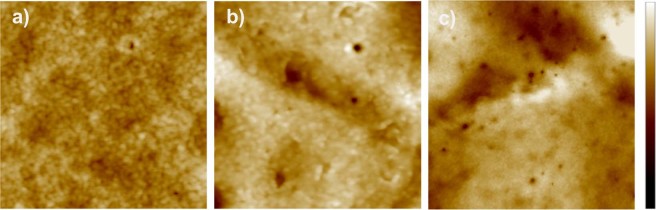
AFM images of the presence of pore-like damages over time in RBCs, (**a**) non-treated, 1 μm × 1 μm, (**b**) 5 min, 1 μm × 1 μm, and (**c**) 30 min, 2 μm × 2 μm; Z bar scale 20 nm.

Additionally, it should be noted that we also observed superficial damages of different nature on cell membranes that could be attributed to PFTs^26^. The presence of these pore-like damages increased over time, as shown in Figs 10b and 11. Nonetheless, while damages attributed to PLA2 activity are observed after 5 min, it is necessary to wait for 30 minutes to visualize significative alterations tentatively caused by PFTs.

## Conclusions

The fire coral, *Millepora alcicornis*, causes hemolytic effects upon contact attributed to the biosynthesis of proteins of the phosholipase A2 (PLA2) type as a main defense mechanism. These proteins rapidly catalyse the hydrolysis at the sn-2 position of the glycerol moiety of phosphatidylcholine-type phospholipids of cellular membranes. The hydrolysis reaction triggers a structural damage in the outer leaflet of the lipidic bilayer of membranes by generating pores that lead the cellular content to flow out of the damaged cells and depletion. All this sequence of events clearly shows the mode of action of PLA2 proteins on the structure of red blood cells. Additionally, we have observed a secondary mechanism tentatively caused by pore-forming protein toxins (PFTs). With the use of atomic force microscopy, the complete hemolysis process could be visualized in detail at the nanoscale level^36^. Nonetheless, we are currently working to unravel the specific mechanisms related with both PLA2s and PFTs of *Millepora alcicornis*.

## Material

### Specimens collection

The fragments of *Millepora alcicornis* were collected in September, 2018 at El Porís locality, Tenerife Island (28°10′24.12″N, 16°25′47.12″W) where this species was first recorded in 2008. A total of 431 g of different colonies was collected by scuba diving between 6–8 m deep and transported to laboratory in dark and wet conditions where was stored at −20 °C for later use in protein extraction.

### Extraction and preparation of protein extract

Nematocyst discharge was induced by stirring coral fragments in deionized water at 4 °C for 24 hours. The extract obtained was centrifuged at 3000 rpm for 15 min at 4 °C using a Thermo Scientific Sorvall RC6 + centrifuge (F14S-6 × 250X rotor). This procedure was repeated twice, and the supernatant was freeze-dried and stored at −20 °C^16^.

### Protein concentration determination

The lyophilized protein extract (150 mg/mL) was dissolved in deionized water and filtered through a PTFE- membrane 0.45 μm (Minisart SRP 15, Sartorius Stedim Biotech GmbH) and quantification of protein content was determined by the Bradford method, using a standard curve prepared with lyophilized bovine serum albumin (Sigma-Aldrich)^16^.

### Ethical statement

All animal procedures were performed in accordance guidelines and regulations approved by the Ethical Commission of University of La Laguna (ULL). This study was completed in strict accordance with the authorization and protocol reference number ULL-CEIBA2015-0171.

### Red blood cells

Red blood cells (RBCs) samples were obtained from the coccygeal artery of Sprange Dawley rats anesthetized with isoflurane using vials that containing EDTA as anticoagulant (BD Vacutainer K3E 15%, Aprotinin 250 KIU, Ref. 361017). The samples were kept at 4 °C until their use.

### Hemolytic activity

The hemolytic activity of the aqueous extract of *M*. *alcicornis* was tested by using blood samples from Sprange Dawley rats on 96-well plates. Blood cells washed three times with saline solution were combined with the protein aqueous extract at different concentrations (0.1300, 0.2600, 0.5200, 1.040, 2.080 μg lyophilized extract/mL; equivalent to 0.0088, 0.0177, 0.0353, 0.0707, 0.1413 μg total protein/mL). The samples were incubated at 37 °C for 45 minutes. After centrifugation, the absorbance was measured at 405 nm to determine the hemoglobin released from lysed eritrocytes. Saline solution was used as negative control and TritonX (0.1%) as positive control of hemolysis in the experiment. Results were normalized to 100% hemolysis with positive control. One hemolytic unit (HU_50_) was defined as the amount of protein sample required to cause 50% hemolysis. All experiments were performed three times, and the mean values and standard deviations were also calculated.

### Red blood cell treatment

Incubation: 200 μL of fresh rat blood was incubated with 20 μL of a 1000 μg/mL of protein solution to give a final concentration of 100 μg/mL of protein. After the incubation time (t = 1, 5 and 30 min), 10 μL of the treated blood sample was smeared on glass slides. Samples were dried by 10 min before AFM analysis.

### AFM analysis

AFM topographic images were obtained in Peak Force mode using a multimode microscope with a Nanoscope V control unit from Bruker. Scan rates of 0.5–1.2 Hz and FESP (50–100 kHz, and 1–5 N m-1) tips (from Bruker) were used^37^. To get representative information about the hemolysis process of the RBCs images from (100 µm × 100 µm) to (0.6 µm × 0.6 µm) were recorded using 512 points/line. In addition to the topographic images, the so-called “Peak Force error image” provides a better visualization of the surface features. Error image information can be assumed as the derivative of the topographic image, giving in this way an enhancement of the height differences. AFM images analysis software from Bruker and from Gwyddion were used.

## Supplementary information

Supporting information
