# Supplementary material for: Damages at the nanoscale on red blood cells promoted by fire corals
Source: Sci Rep. 2019 Oct 4;9:14298. doi: 10.1038/s41598-019-50744-6 (PMC6778144; doi:10.1038/s41598-019-50744-6)
Supplement: Supplementary file 1 — Supporting information [file 41598_2019_50744_MOESM1_ESM.pdf]

# Supporting Information

# Damages at the nanoscale on red blood cells promoted by fire corals

**Ana R. Díaz-Marrero[1,†], Miriam C. Rodríguez González[2,†], Alberto Hernández Creus[2], Adriana Rodríguez Hernández[3], José J. Fernández[1,4,*]**

[1] Instituto Universitario de Bio-Orgánica Antonio González (IUBO AG), Centro de Investigaciones Biomédicas de Canarias (CIBICAN), Universidad de La Laguna (ULL), Avda. Astrofísico Francisco Sánchez 2, 38206 La Laguna, Tenerife, Spain
[2] Área de Química Física, Departamento de Química, Instituto de Materiales y Nanotecnología (IMN), Universidad de La Laguna (ULL), Avda. Astrofísico Francisco Sánchez s.n., 38206 La Laguna, Tenerife, Spain
[3] Departamento de Biología Animal, Edafología y Geología. UD Ciencias Marinas. Facultad de Ciencias (Sección Biología), Universidad de La Laguna (ULL), Avda. Astrofísico Francisco Sánchez s.n., 38206 La Laguna, Tenerife, Spain
[4] Departamento de Química Orgánica, Universidad de La Laguna (ULL), Avda. Astrofísico Francisco Sánchez s.n., 38206 La Laguna, Tenerife, Spain
† These authors contributed equally to the work. Correspondence and requests for materials should be addressed to J.J.F. (email: jjfercas@ull.edu.es)

# Table of Contents


| | Contents | Page |
|---|---|---|
| 1.- | **Figures S1-S4.** Colonies of *Millepora alcicornis* and details of the coral in Tenerife Island. | S3-S6 |
| 2.- | **Figure S5.** AFM images of the different RBCs morphologies present on a blood sample (taken from a 100 µm x 100 µm): **a**) discocytes, **b)** stomatocytes; **c)** echinocytes and **d)** planocytes. Cross sections of the RBCs are included in order to observe better their profiles. | S7 |
| 3.- | **Figure S6.** AFM images for untreated red blood cells (CRBs). | S8 |
| 4.- | **Figures S7-S9.** AFM images of the RBCs: Top view and error signal for a cell treated at 1 min with initial damages at the lipid bilayer and details. | S9-S11 |
| 5.- | **Figures S10-S12.** AFM images of the RBCs: Top view and error signal for a cell treated at 5 min. Membrane damages at the lipid bilayer and pores formation. | S12-S14 |
| 6.- | **Figures S13-S15.** AFM images of the RBCs: Top view and error signal for a cell treated at 10 min. Membrane with a large lipid bilayer affectation. | S15-S17 |
| 7.- | **Figure S16-17.** AFM images of the RBCs: Top view image for empty cells treated at 30 min and cross section | S18-S19 |
| 8.- | **Figure S18.** AFM images of **a)** 90 µm x 90 µm and **b)** 7 µm x 7 µm of the RBCs after 5 minutes in contact with ultrapure Milli-Q water. | S20 |

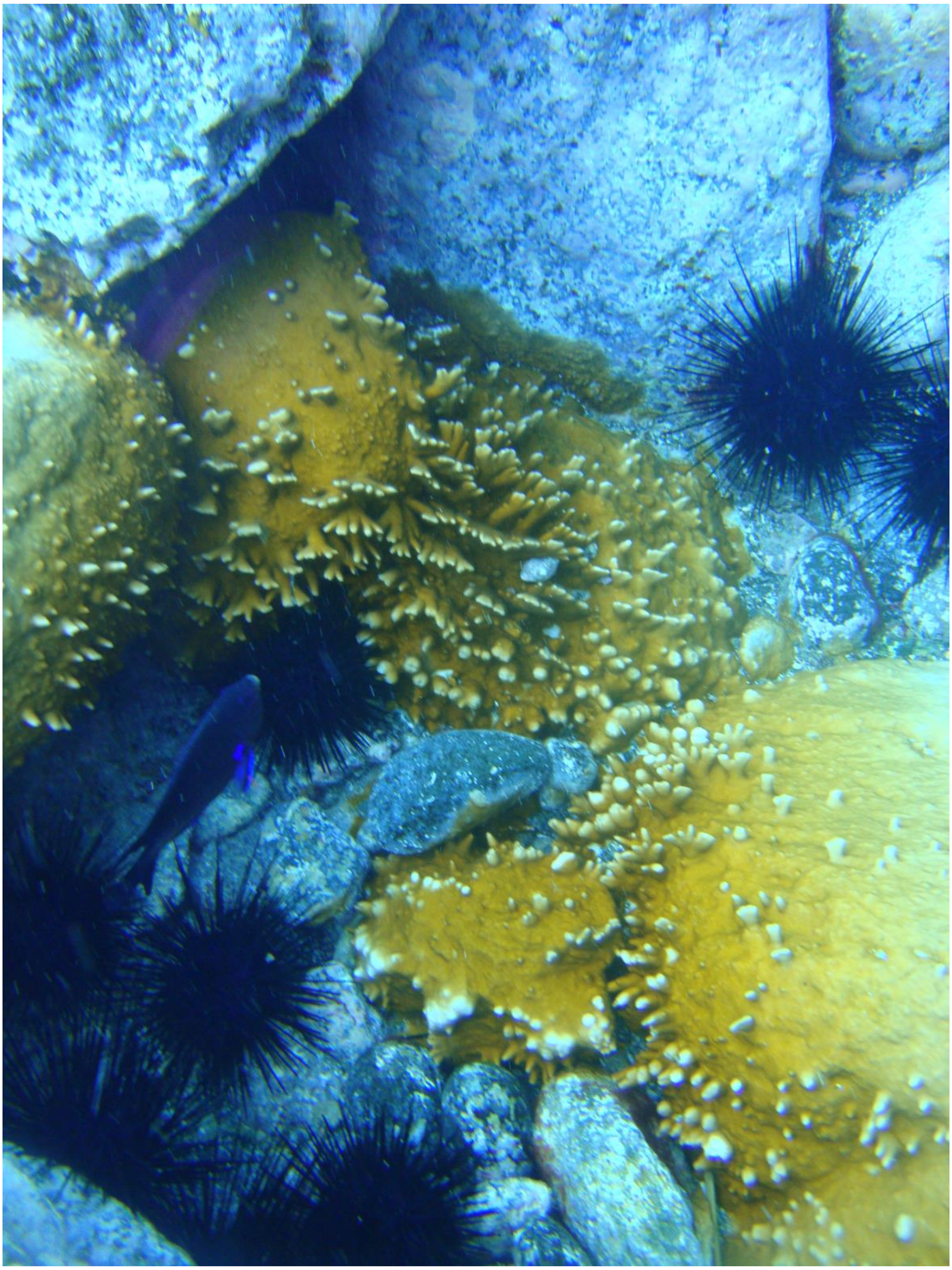

**Figure S1.** Marine ecosystem with colonies of *Millepora alcicornis* in Tenerife Island.

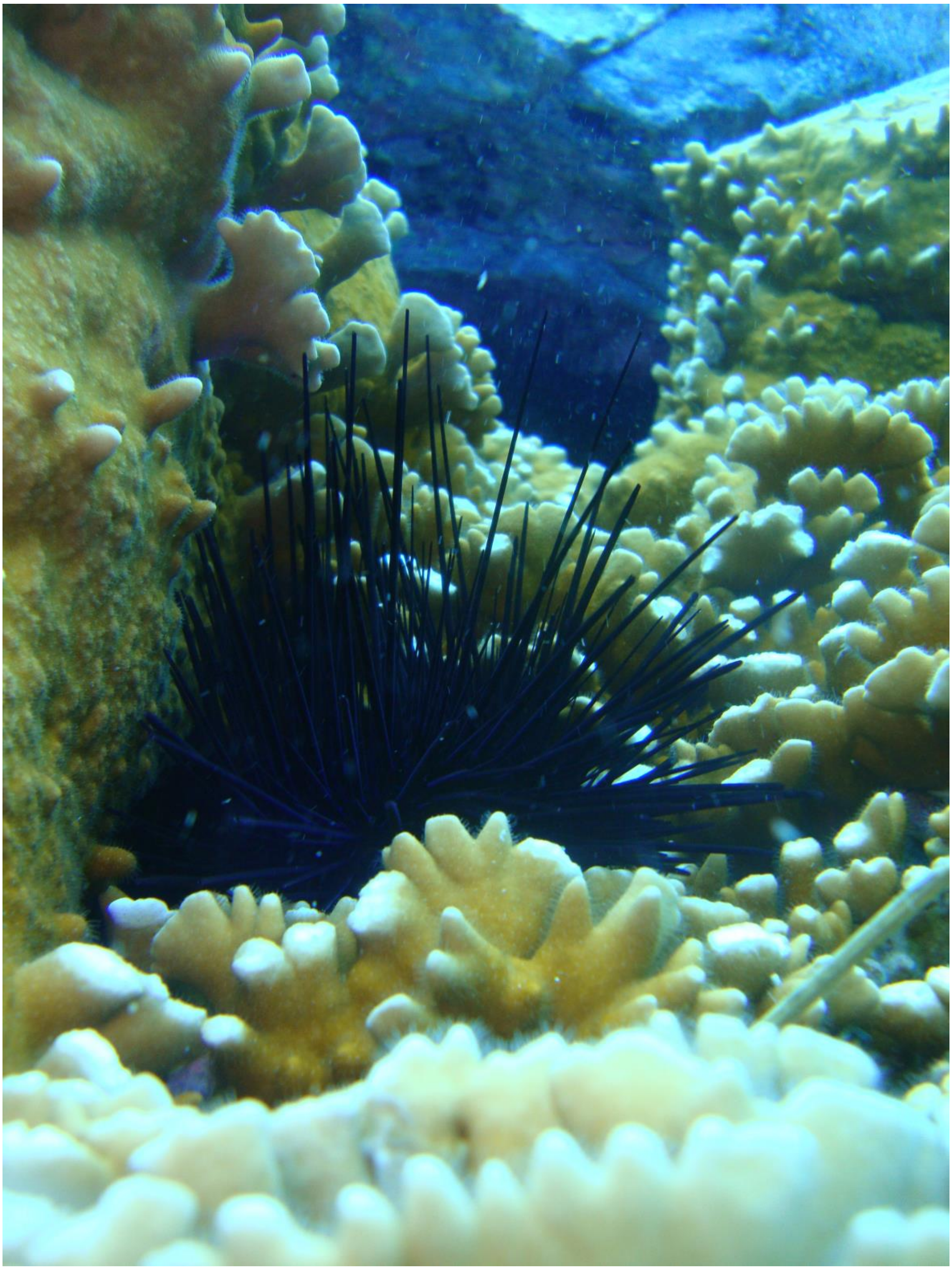

**Figure S2.** Colonies of *Millepora alcicornis* in Tenerife Island.

.

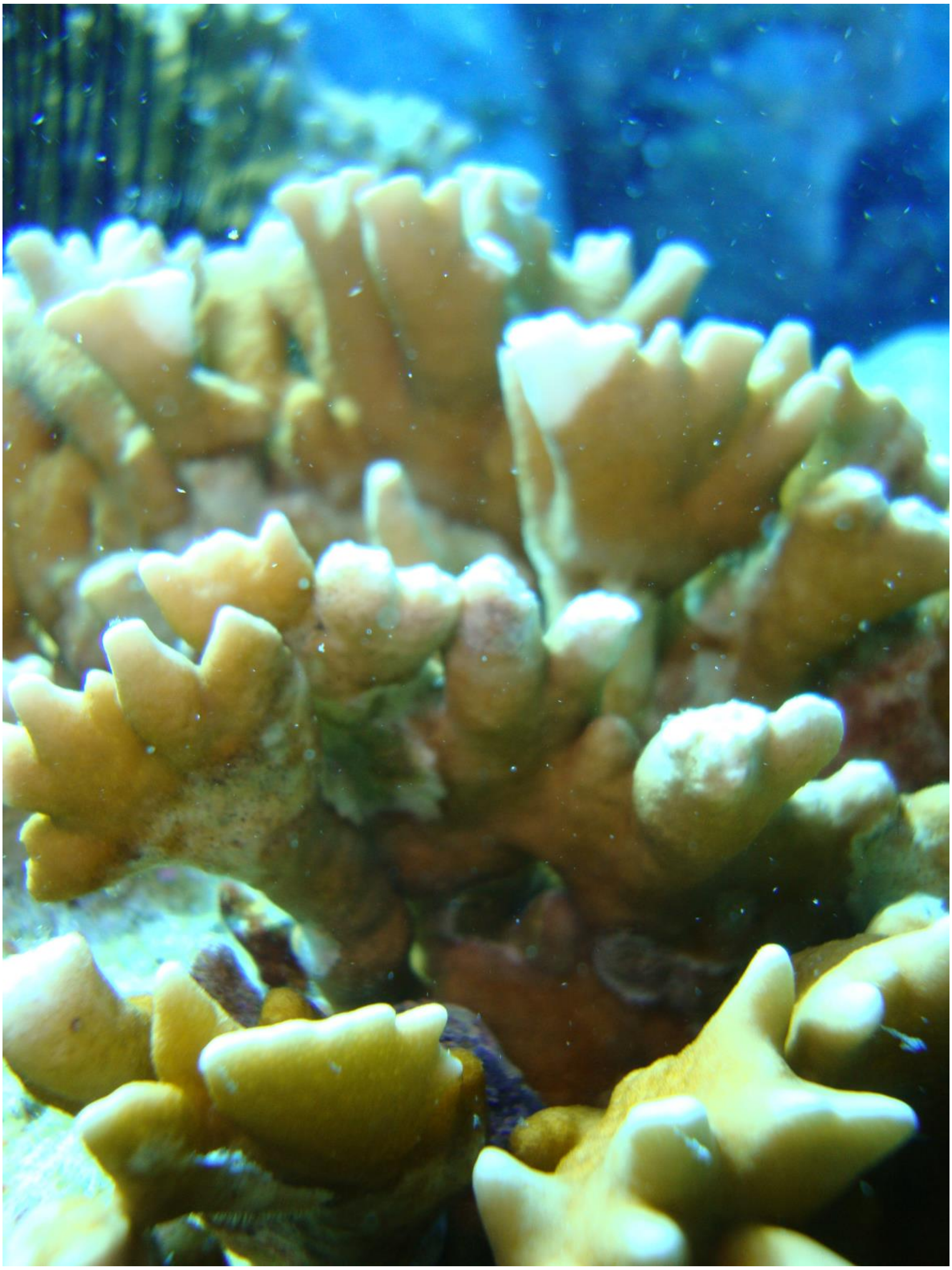

**Figure S3.** Detail of *Millepora alcicornis* in Tenerife Island.

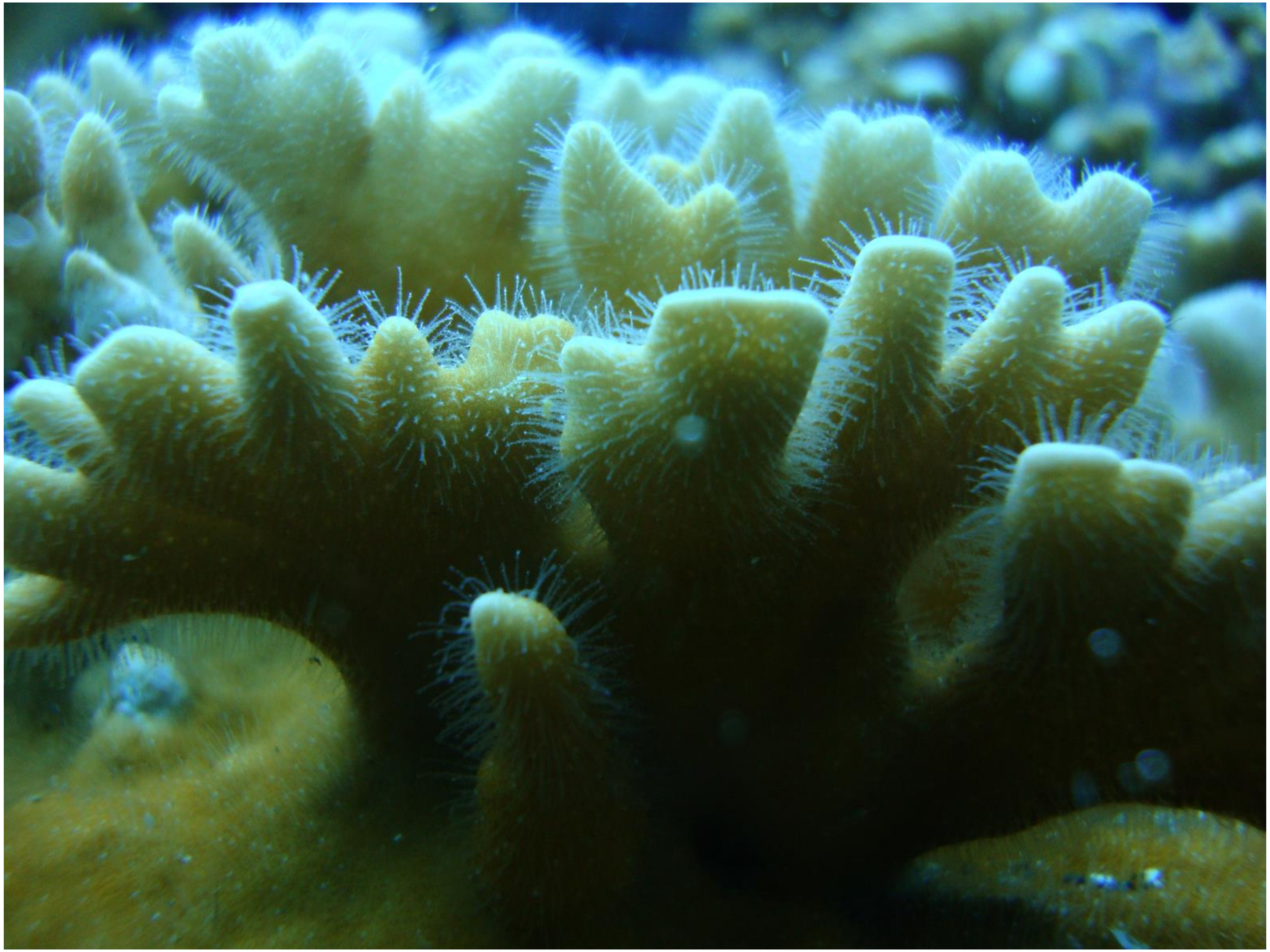

**Figure S4.** Detail of *Millepora alcicornis* in Tenerife Island.

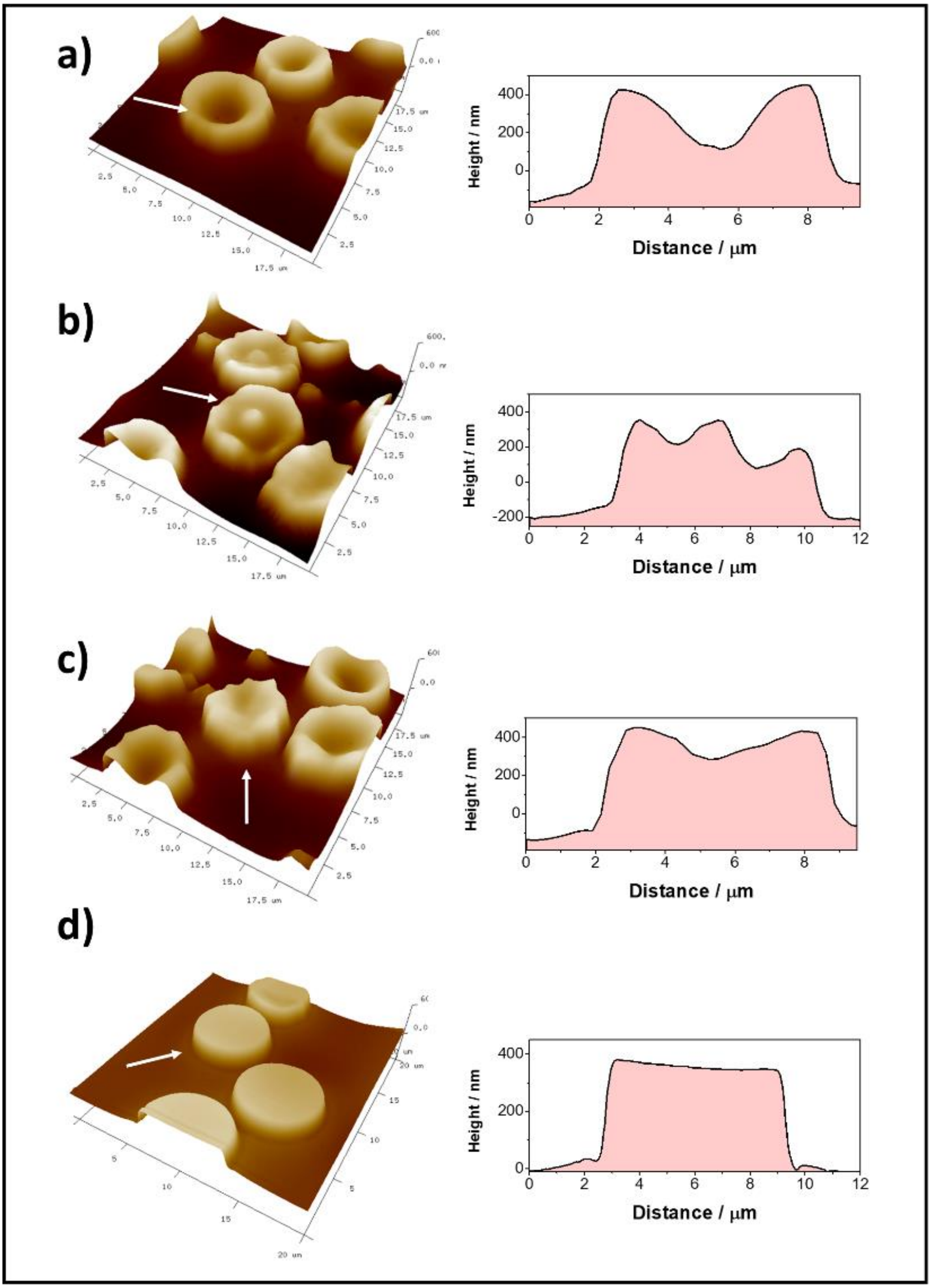


**Figure S5.** AFM images of the different RBCs morphologies present on a blood sample (taken from a 100 µm x 100 µm): a) discocytes, b) stomatocytes; c) echinocytes and d) planocytes. Cross sections of the RBCs are included in order to observe better their profiles.

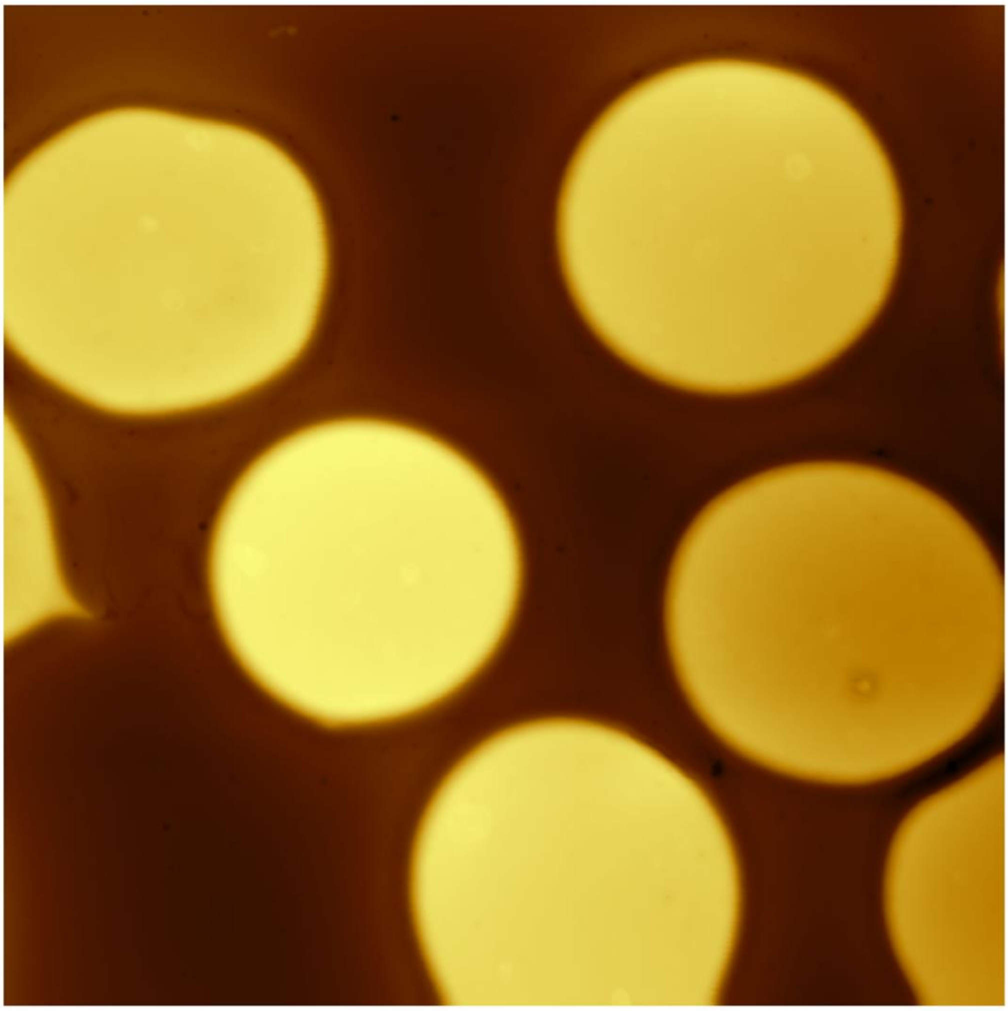

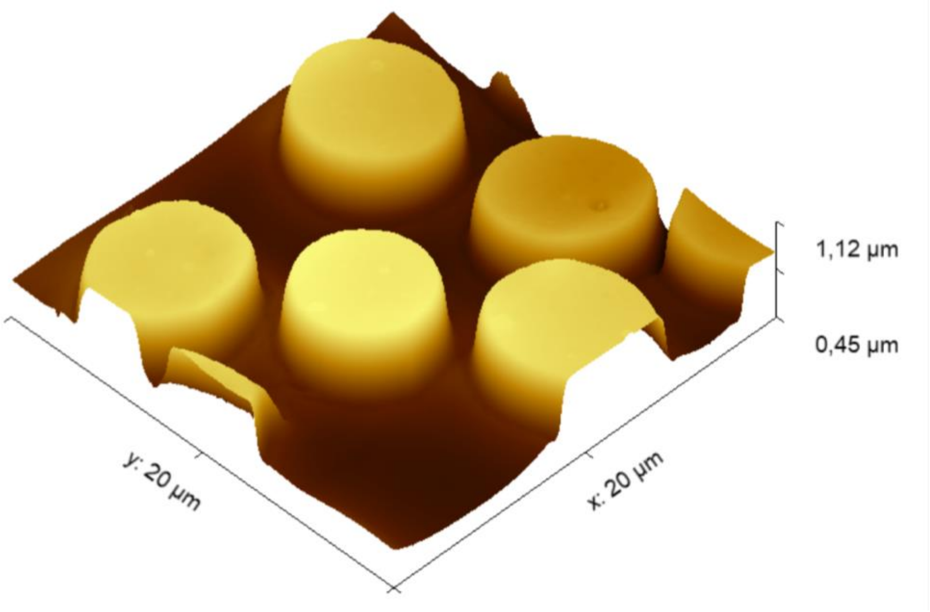


**Figure S6.** AFM images of the RBCs: Top view and 3D AFM images of 20 µm x 20 µm of untreated cells.

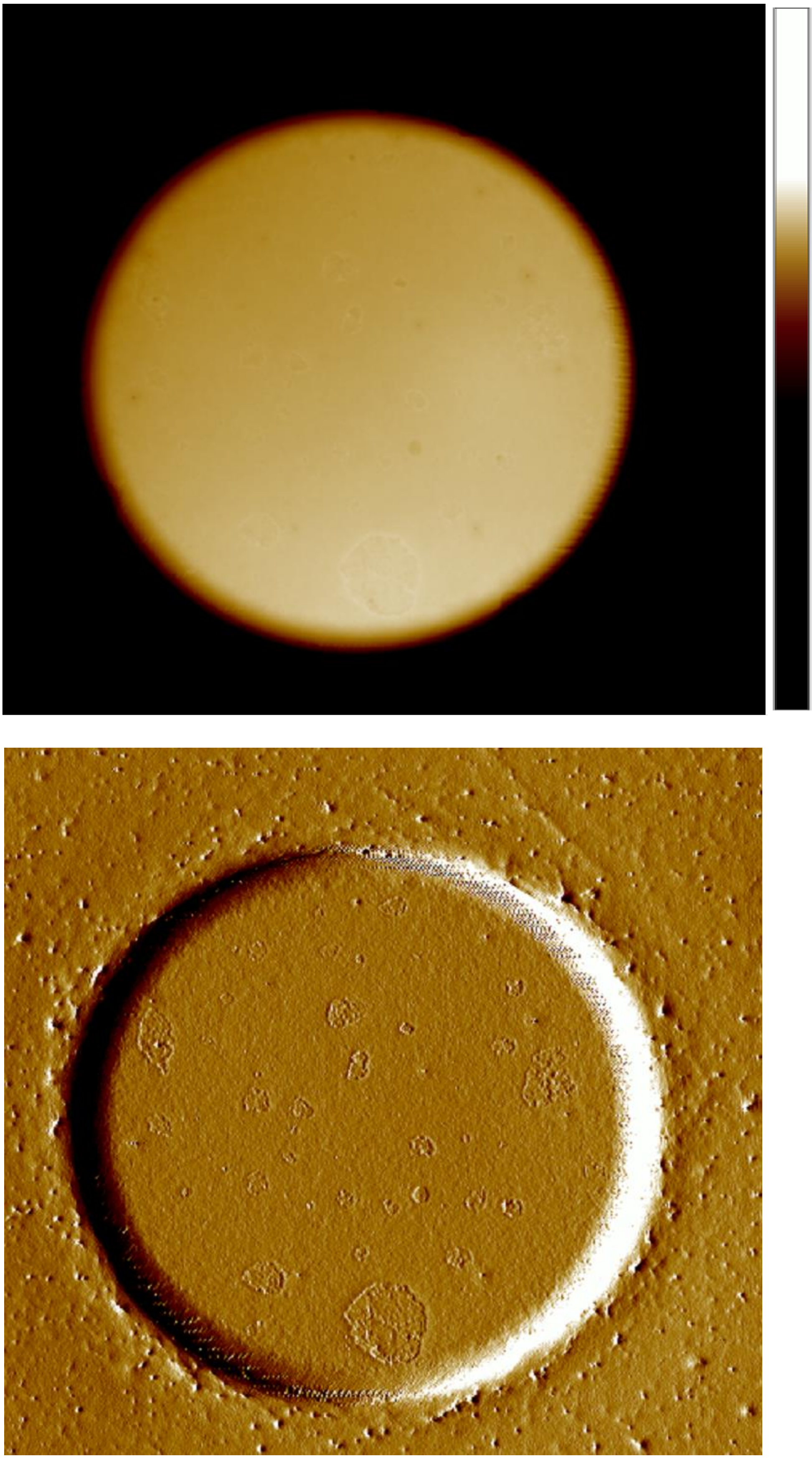

**Figure S7.** AFM images of RBCs: Top view (9.2 µm x 9.2 µm, Z bar scale 1 µm) and error signal (bottom). Cell treated at 1 min with initial damages at the lipid bilayer.

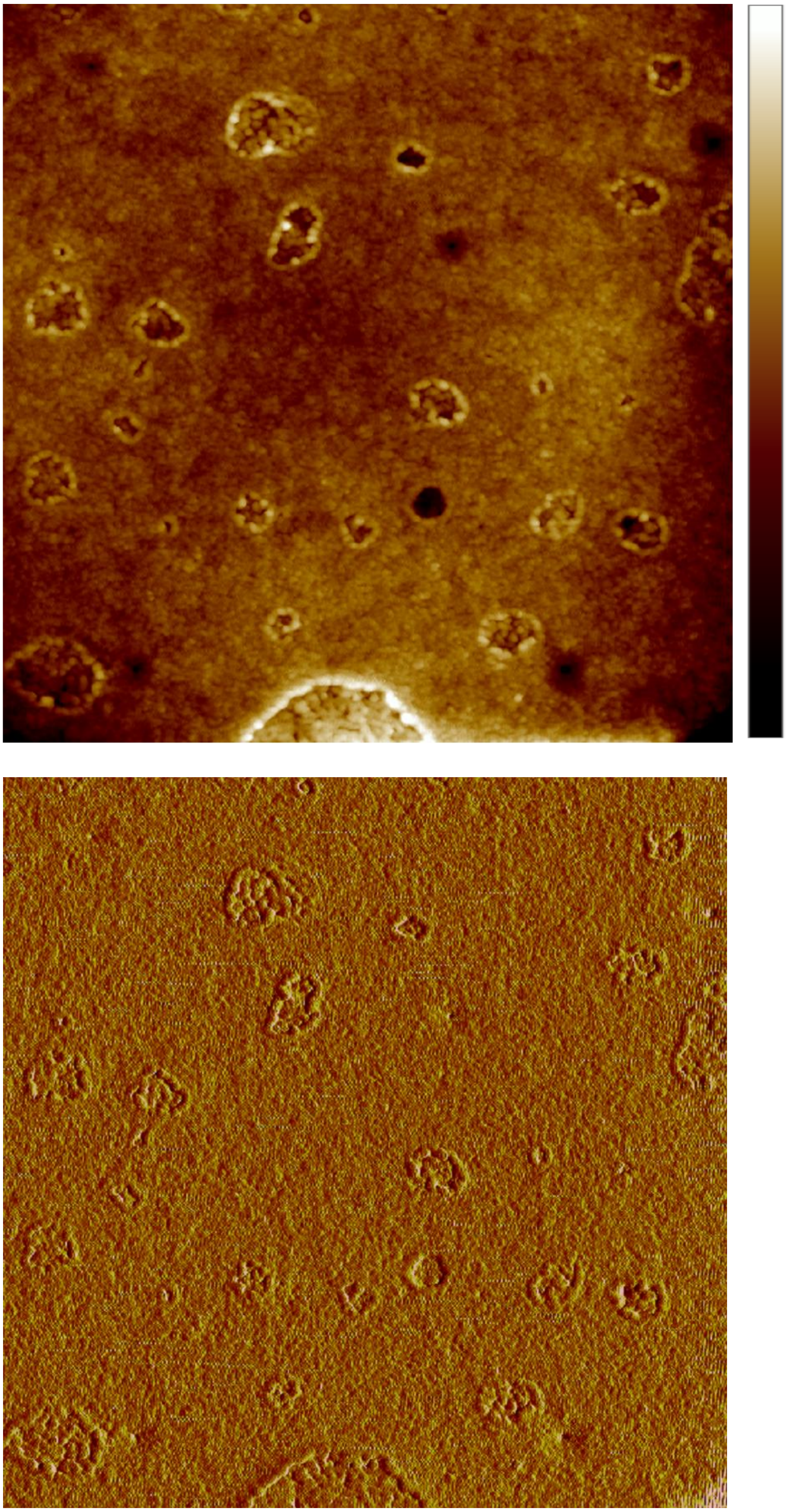

**Figure S8.** AFM images of RBCs: Top view (3.6 µm x 3.6 µm, Z bar scale 20 nm) and error signal (bottom). Cell treated at 1 min. Detail of the initial damages at the lipid bilayer.

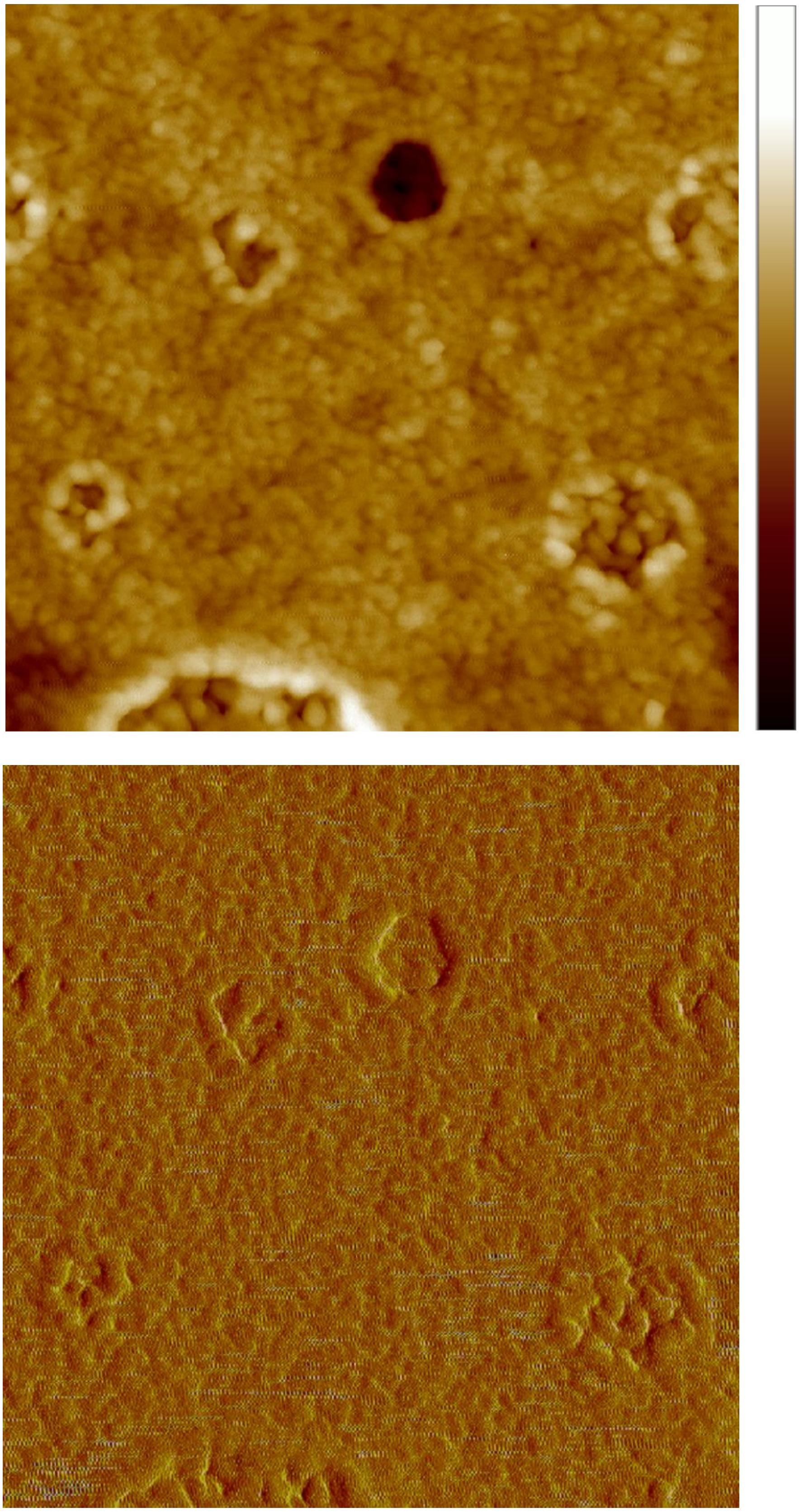

**Figure S9.** AFM images of RBCs: Top view (1.5 µm x 1.5 µm, Z bar scale 20 nm) and error signal (bottom). Cell treated at 1 min. Detail of the initial damages at the lipid bilayer pore formation.

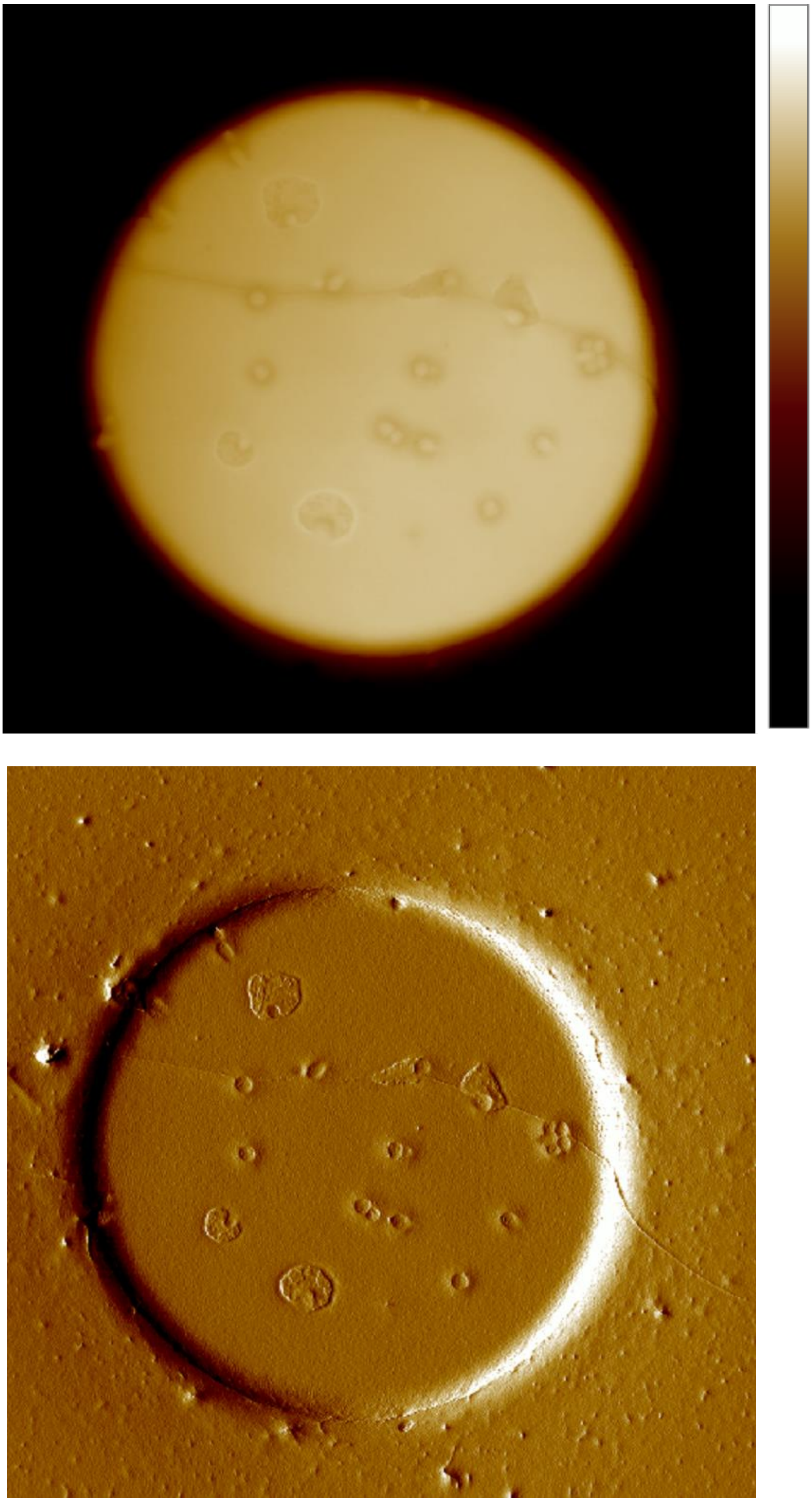

**Figure S10.** AFM images of RBCs: Top view (10 µm x 10 µm, Z bar scale 500 nm) and error signal (bottom). Cell treated at 5 min. Progress in damages at cell membrane**.**

.

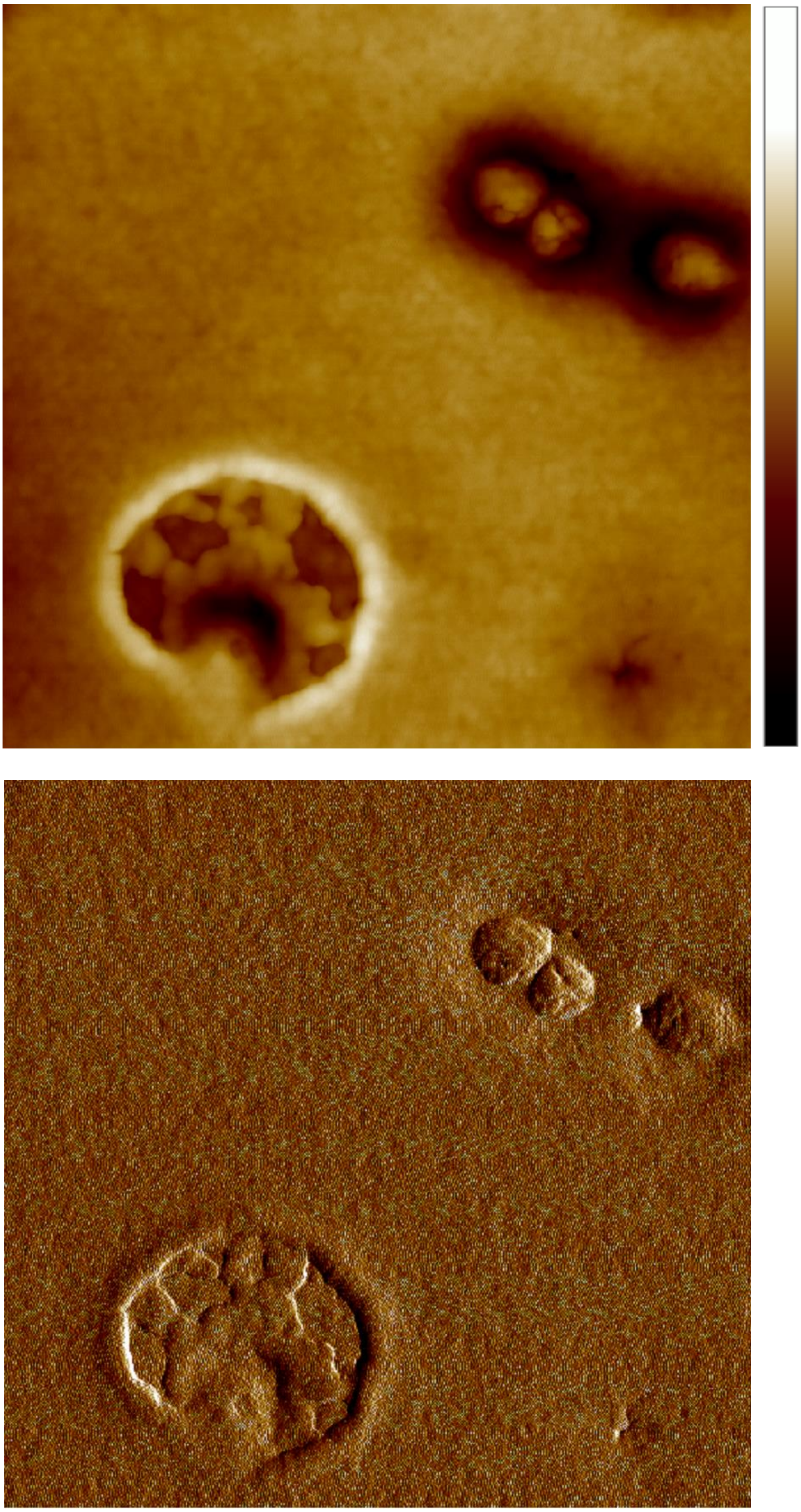

**Figure S11.** AFM images of RBCs: Top view (2 µm x 2 µm, Z bar scale 40 nm) and error signal (bottom). Cell treated at 5 min. Detail of the cell membrane perforation.

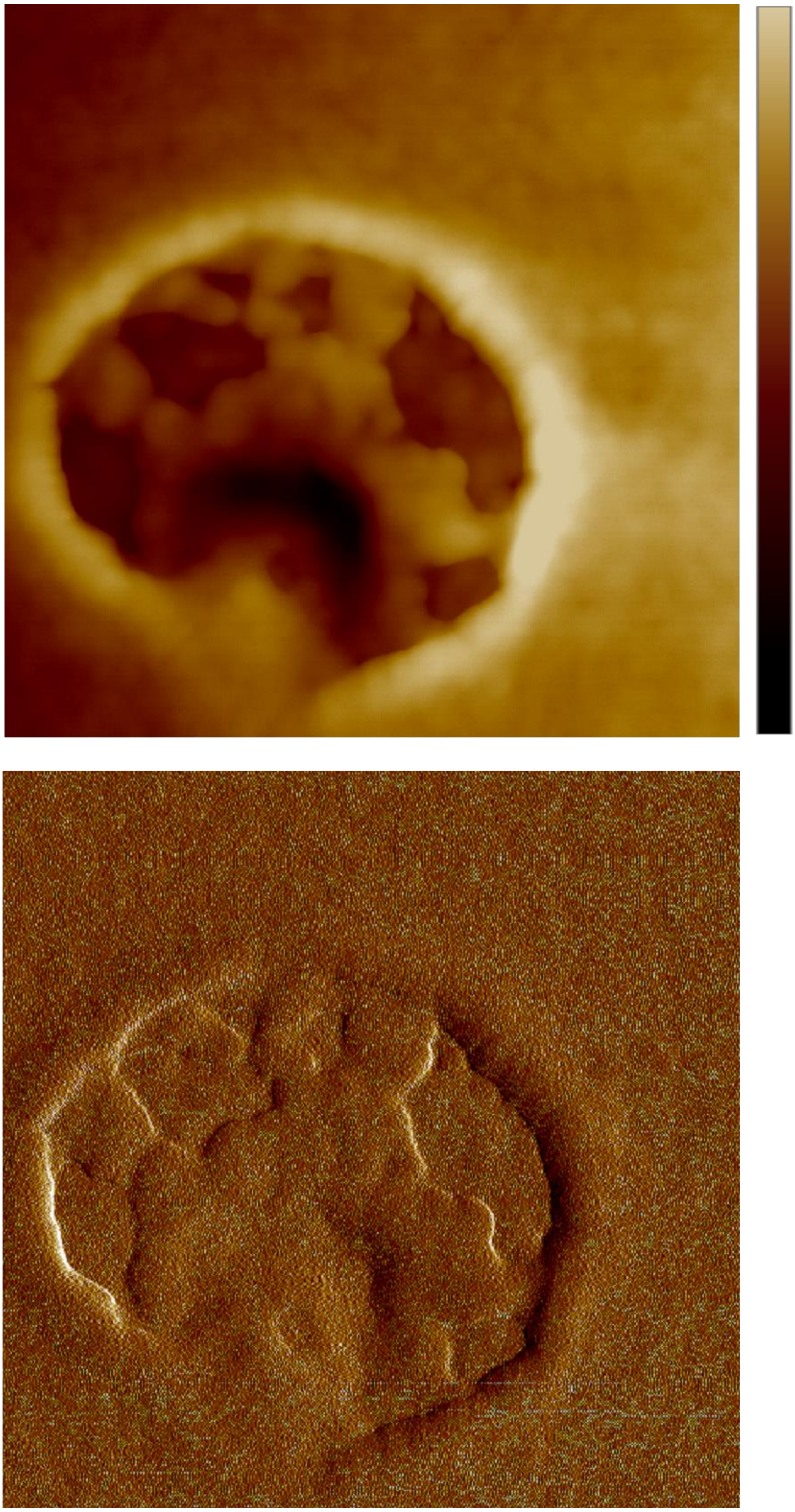

**Figure S12.** AFM images of RBCs: Top view (1 µm x 1 µm, Z bar scale 30 nm) and error signal (bottom). Cell treated at 5 min. Detail of the cell membrane perforation.

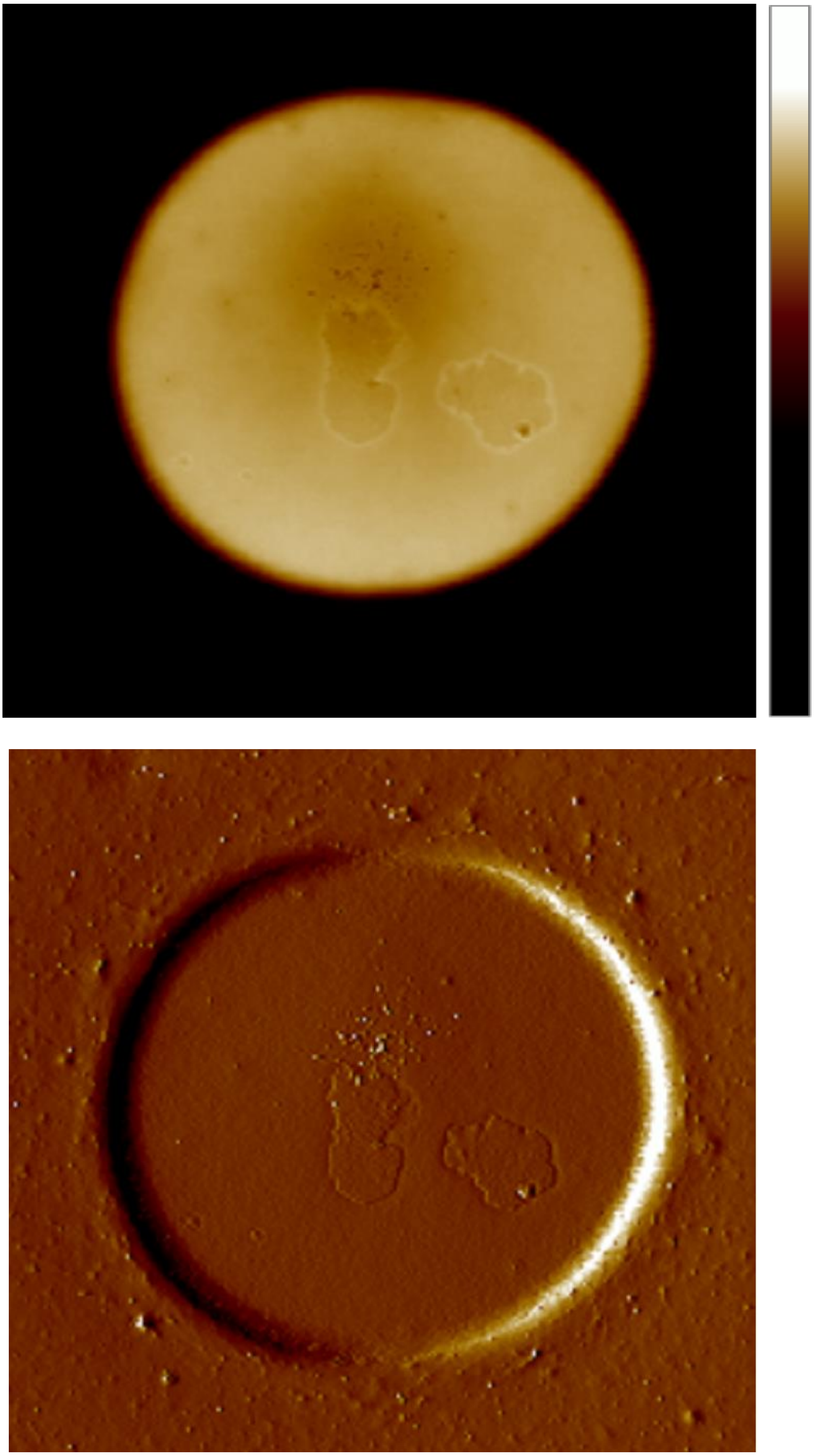

**Figure S13.** AFM images of RBCs: Top view (11.3 µm x 11.3 µm, Z bar scale 400 nm) and error signal (bottom). Cell treated at 10 min. Cell with strong membrane affectation.

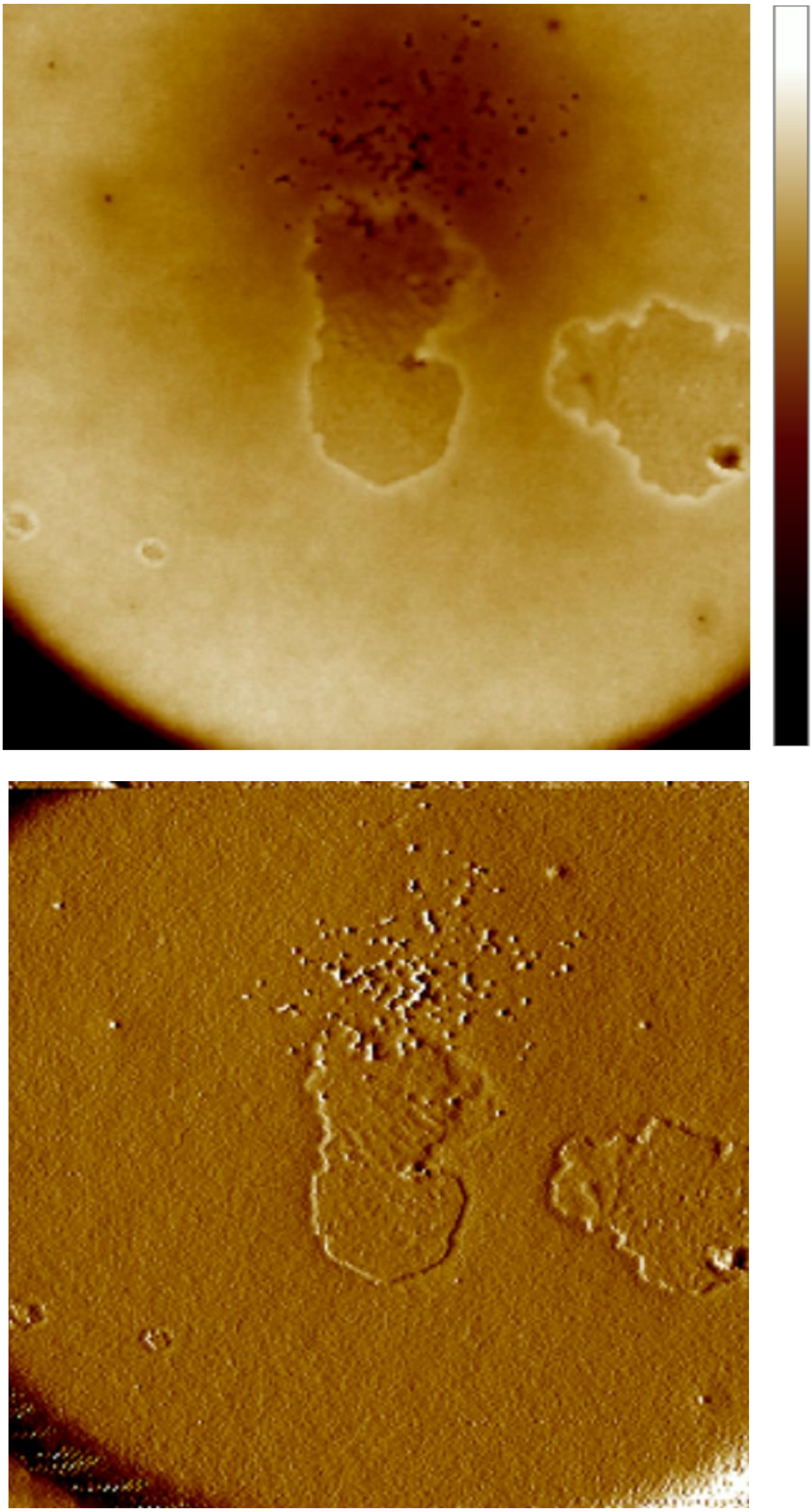

**Figure S14.** AFM images of RBCs: Top view (4.7 µm x 4.7 µm, Z bar scale 100 nm) and error signal (bottom). Cell treated at 10 min. Detail of cell membrane injuries.

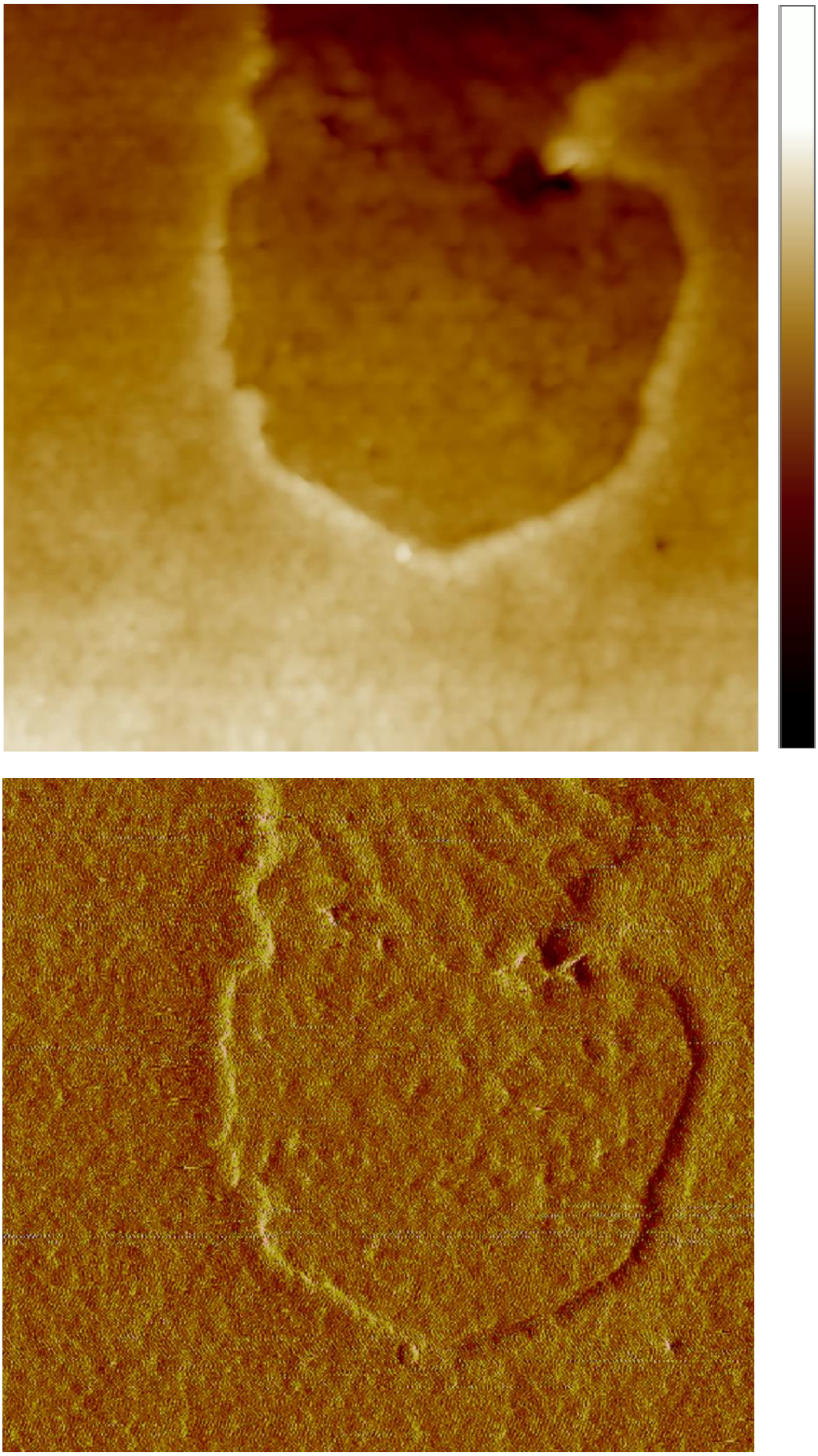

**Figure S15.** AFM images of RBCs: Top view (1.6 µm x 1.6 µm, Z bar scale 50 nm) and error signal (bottom). Cell treated at 10 min. Detail of cell membrane injuries.

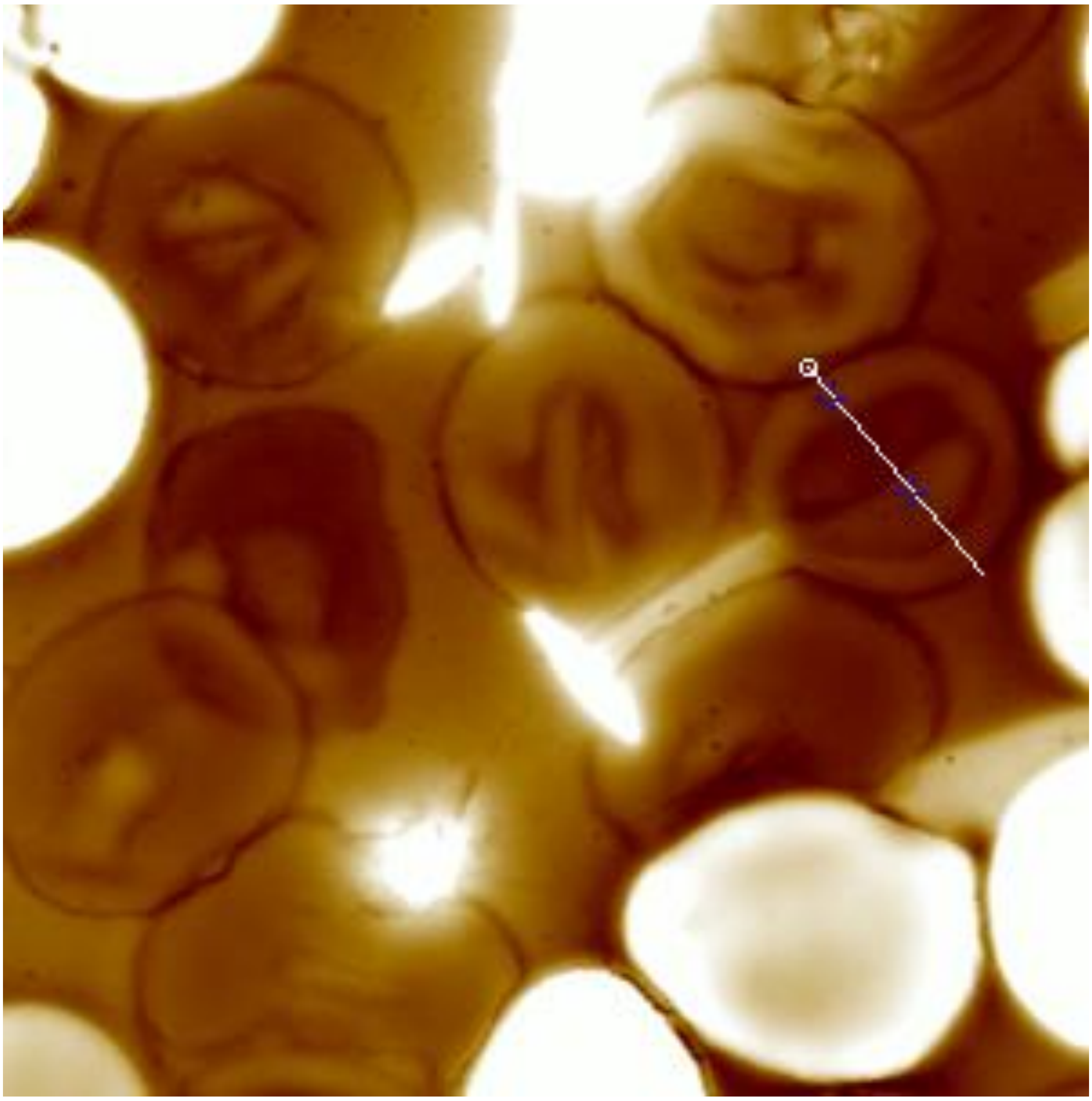

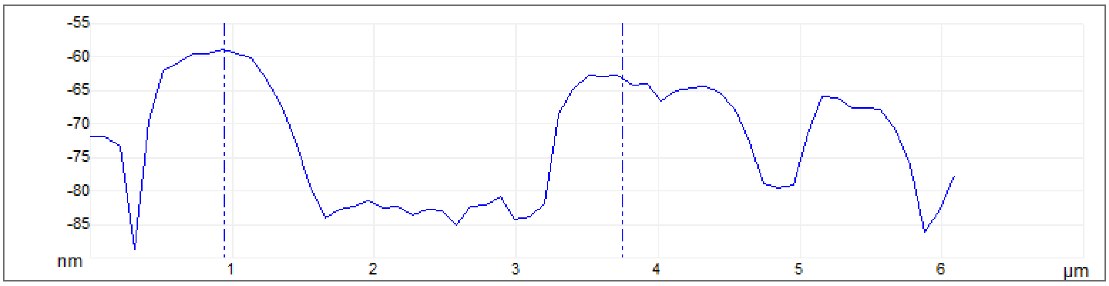


**Figure S16.** AFM images of the RBCs: Top view image for empty cells treated at 30 min and cross section

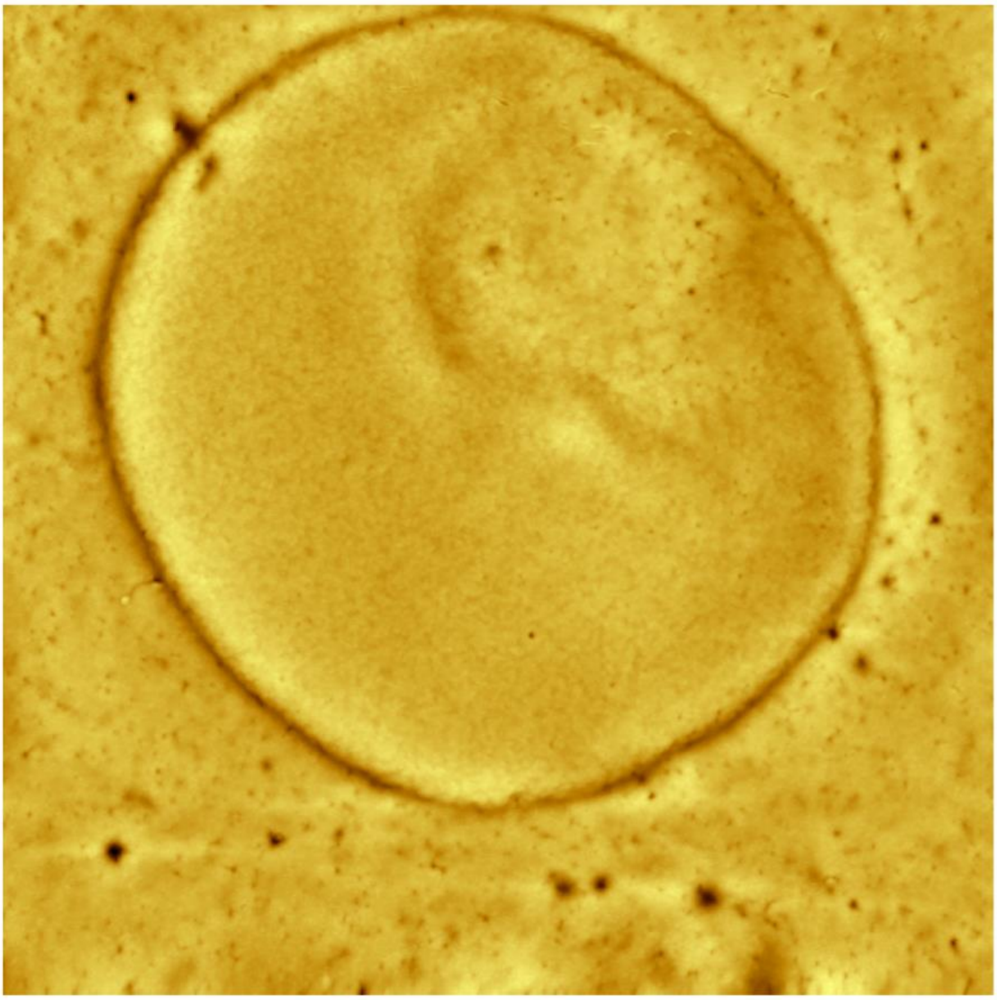

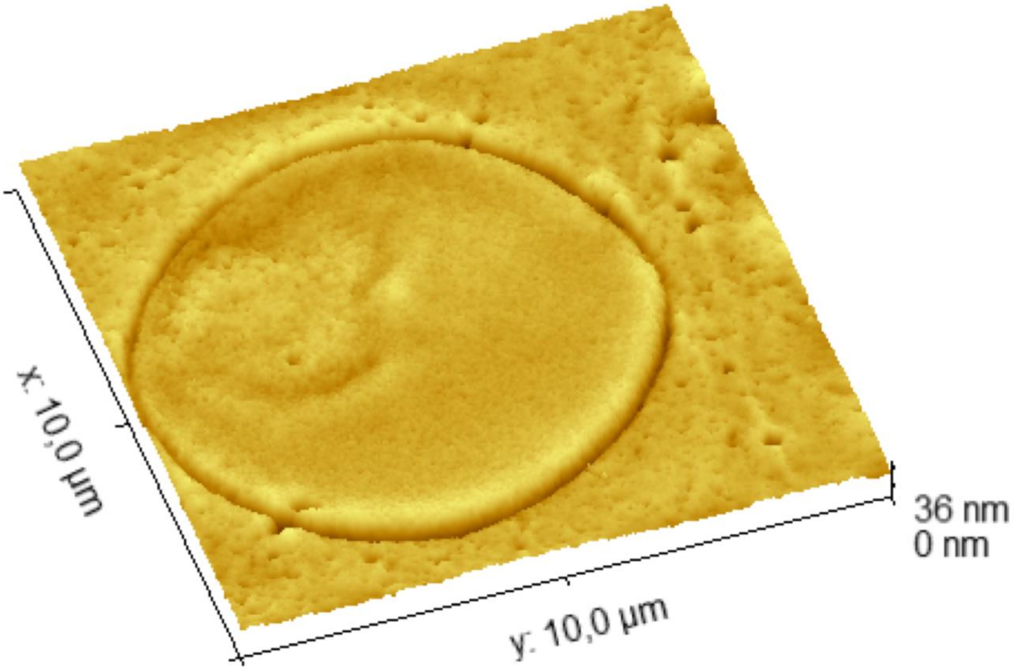


**Figure S17.** AFM images of the RBCs: Image for empty cells treated at 30 min.

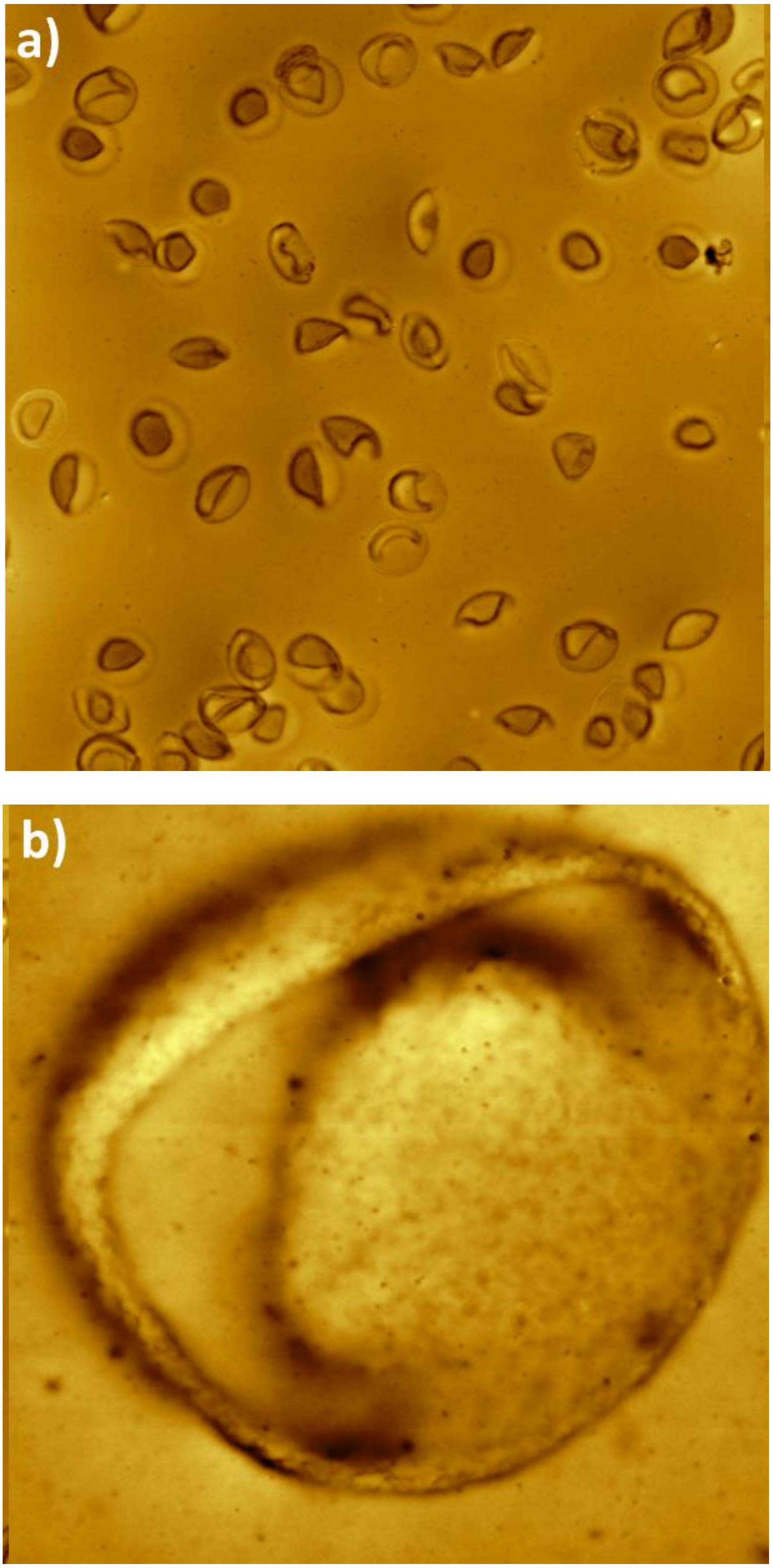


**Figure S18.** AFM images of a) 90 µm x 90 µm and b) 7 µm x 7 µm of the RBCs after 5 minutes in contact with ultrapure Milli-Q water.
